# Distinct carbon sources affect structural and functional maturation of cardiomyocytes derived from human pluripotent stem cells

**DOI:** 10.1038/s41598-017-08713-4

**Published:** 2017-08-17

**Authors:** Cláudia Correia, Alexey Koshkin, Patrícia Duarte, Dongjian Hu, Ana Teixeira, Ibrahim Domian, Margarida Serra, Paula M. Alves

**Affiliations:** 1 iBET, Instituto de Biologia Experimental e Tecnológica, Apartado 12, Oeiras, 2780-901 Portugal; 2 Instituto de Tecnologia Química e Biológica António Xavier, Universidade Nova de Lisboa (ITQB-NOVA), Oeiras, 2780-157 Portugal; 3 Cardiovascular Research Center, Massachusetts General Hospital, Boston, MA 02114 USA; 4 Harvard Medical School, Boston, MA 02115, USA, Harvard Stem Cell Institute, Cambridge, MA 02138 USA; 5 ETH Zurich, Department of Biosystems Science and Engineering, Mattenstrasse 26, 4058 Basel, Switzerland

## Abstract

The immature phenotype of human pluripotent stem cell derived cardiomyocytes (hPSC-CMs) constrains their potential in cell therapy and drug testing. In this study, we report that shifting hPSC-CMs from glucose-containing to galactose- and fatty acid-containing medium promotes their fast maturation into adult-like CMs with higher oxidative metabolism, transcriptional signatures closer to those of adult ventricular tissue, higher myofibril density and alignment, improved calcium handling, enhanced contractility, and more physiological action potential kinetics. Integrated “-Omics” analyses showed that addition of galactose to culture medium improves total oxidative capacity of the cells and ameliorates fatty acid oxidation avoiding the lipotoxicity that results from cell exposure to high fatty acid levels. This study provides an important link between substrate utilization and functional maturation of hPSC-CMs facilitating the application of this promising cell type in clinical and preclinical applications.

## Introduction

In the last decade remarkable progress has been made towards the establishment of protocols for directed differentiation of human pluripotent stem cells (hPSCs, including hiPSCs and hESCs) into cardiomyocytes (hPSC-CMs)^1^. However, hPSC-CMs are often immature, showing metabolic, structural and functional characteristics that more closely resemble fetal CMs rather than adult CMs^2^. hPSC-CMs commonly display disarrayed sarcomeres, irregular shapes, underdeveloped mitochondria and use glucose (Glc) as major energy source, contrasting with adult CMs which present organized sarcomere structures, rod-shaped morphologies, well-developed mitochondria with mature lamellar cristae and rely on fatty acid (FA) β-oxidation for energy production^2, 3^.

Although some efforts have been made recently towards the development of methods for enhancing hPSC-CM maturity (by increasing time in culture^4^, applying mechanical and electrical stimulation^5–7^, adding chemicals or small molecules^8^, adjusting substrate stiffness^9^, using genetic approaches^10, 11^ or growth as 3-dimensional (3D) tissues^12–15^) the outcomes have been variable. The use of distinct sets of analyses for CM maturation profile characterization has also limited the direct comparison between different studies.

CM maturation *in vivo* has been associated with a transition from an embryonic-like glycolytic to an adult-like oxidative metabolism^16^. In a normal heart, 70% of ATP generation comes from FA oxidation, whereas Glc, lactate (Lac) and pyruvate (Pyr) provide only 30% of the energy produced^17^. It has been shown that hPSC-CMs that are metabolically dependent on FA β-oxidation, *in vitro*, recapitulate cellular activities of more mature CMs, constituting a reliable model to study late onset disorders^18^. Wen and colleagues were able to unveil the pathological phenotype associated with arrhythmogenic right ventricular dysplasia after inducing metabolic maturation in hiPSC-CMs^19^. The authors used a combination of insulin, dexamethasone and 3-isobutyl-1-methil-xanthine to increase FA synthesis and trigger the activation of PPARα, which led to enhanced mitochondrial oxidative phosphorylation. Although the use of medium additives has proven to induce hiPSC-CM bioenergetics switching, the impact of key medium substrates on modulating hPSC-CM metabolism and maturation profile still need to be investigated.

**Figure 1:**
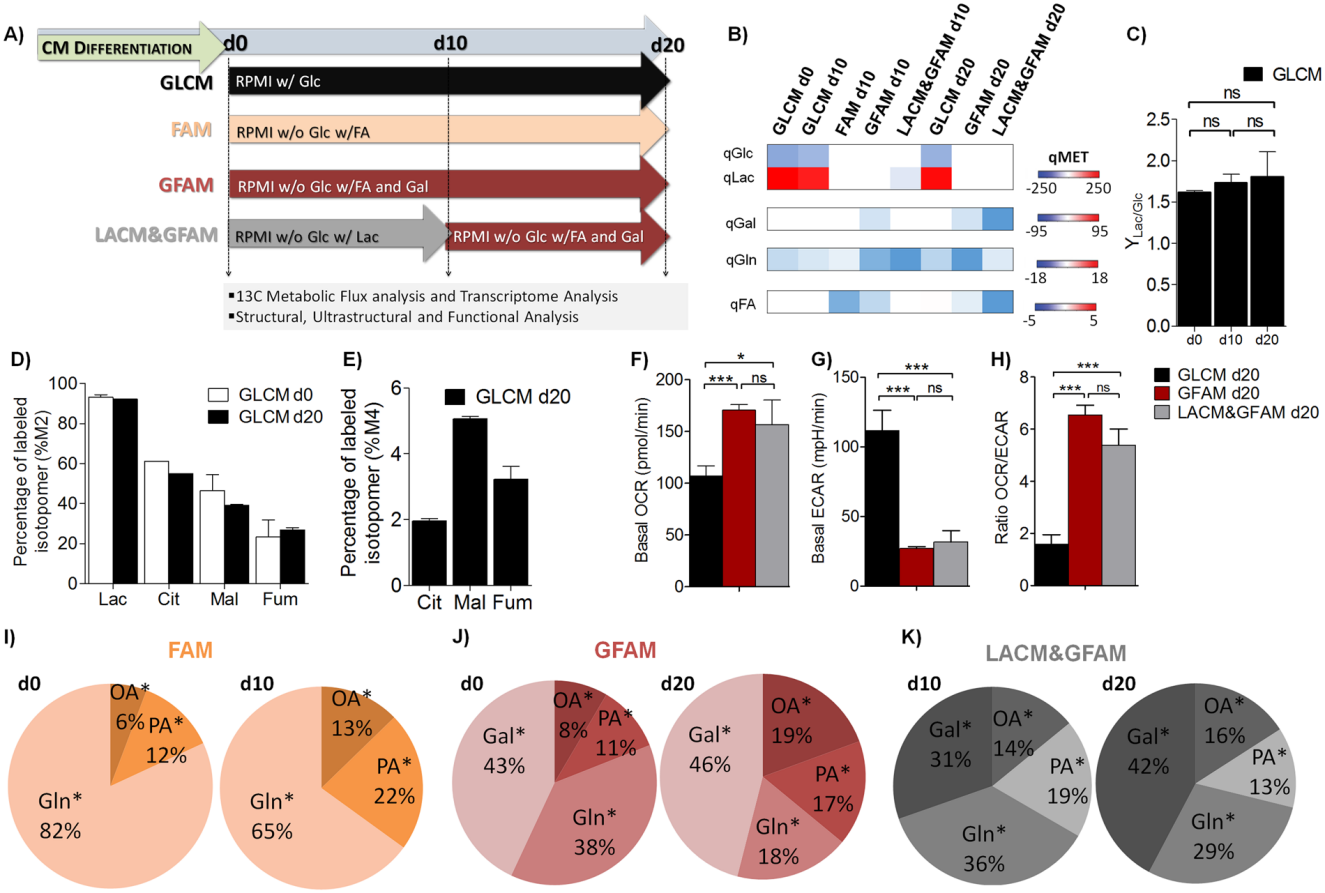
Effect of culture medium composition on central carbon metabolism of hiPSC-CMs. (**A**) Representative scheme illustrating the experimental set-up. The metabolome of hiPSC-CMs was evaluated before (d0) and after 10 (d10) and 20 days (d20) of culture in different media: GLCM (black), FAM (Orange), GFAM (Red) and LACM&GFAM (Grey). (**B**) Heatmap image of metabolome data illustrating specific consumption (blue) and production (red) rates of metabolites (qMET; nmol/(10^6^cell.h)). (**C**) Ratio of Lac production to Glc consumption (YLac/Glc) in GLCM through culture time. (**D**) Percentage of labelled M2 isotopomers from [1,2-^13^C]Glc in Lac and in TCA intermediates (Cit, Fum, Mal). (**E**) Percentage of labelled M4 isotopomers from [U-^13^C]Gln in TCA intermediates. M2 and M4, reflect the first round of TCA cycling of [1,2-^13^C]Glc and [U-^13^C]Gln, respectively. (**F**) Basal oxygen consumption rate (OCR). (**G**) Extracellular acidification rate (ECAR). (**H**) OCR/ECAR ratio reflecting the relative contribution of OXPHOS over glycolysis for energy generation. (**I**–**K**) Pie charts indicating the contribution of the main substrates of each media, for TCA cycle pools. The percentages were determined based on the incorporation of each labelled substrate in Cit, Fum and Mal. OA: Oleic Acid and PA: Palmitic acid. Data are represented as mean ± SD of 12–24 wells, n ≥ 3 separate experiments. *p < 0.05; ***p < 0.001; ns, not significant.

In this study, we hypothesized that manipulating the composition of the hPSC-CM culture medium to mimic the metabolic substrate usage by human adult CMs *in vivo* would induce a glycolytic-to-oxidative metabolic shift and ultimately improve hPSC-CM maturation *in vitro*. We selected multiple medium formulations from the literature^13, 20, 21^ based on the fact that during cardiac development CMs start to use first Lac and then FA as major sources of energy^16^. Specifically, we tested to culture hiPSC-CMs for 20 days in Glc depleted medium supplemented either with: i) FA (FAM), ii) galactose (Gal) and FA (GFAM) or iii) Lac during the first 10 days and then with Gal and FA for the remaining period (LACM&GFAM), using standard Glc-rich hiPSC-CM maintenance medium (GLCM) as reference (Fig. 1A). We investigated for the first time to our knowledge: i) how these different metabolic substrates affect the intracellular metabolism and contribute for the generation of energy; ii) which substrates are more effective in shifting CM metabolism from glycolysis to OXPHOS; iii) if these substrates are triggering molecular, structural and functional maturation of hPSC-CM *in vitro*. By integrating transcriptome, metabolome and fluxome profiling, with an exhaustive structural and functional characterization we provide not only a quantitative dissection of hiPSC-CM phenotypes observed during culture in these different media but also relevant insights on the interaction of CM metabolism and maturation. Importantly, we﻿ demonstrated that GFAM is the best feeding strategy to culture hPSC-CMs, significantly improving several structural and functional maturation features.

## Methods

### hPSC maintenance and differentiation to CMs

In this study two hPSC lines were used namely, the hiPSC line DF19-9-11T.H (WiCell) and the hESC line (HES3 reporter line NKX2-5(eGFP/w)^22^) kindely provided by Dr. David Elliott. hPSCs were routinely expanded in Synthemax II-SC (corning) coated plates in mTESR1 medium (STEMCELL Technologies). Before cardiac differentiation induction, hPSCs were replated on Matrigel® (Corning) coated plates and cultured in mTESR1 medium until reaching 80%-90% confluency. Briefly, expansion medium was replaced by RPMI Medium (ThermoFisher Scientific) supplemented with B27 without insulin (RPMI/B27, ThermoFisher Scientific), 12 µM CHIR99021 (Biogen Cientifica SL), 80 ng/mL Activin A (Tebu-bio) and 50 µg/mL Ascorbic acid (Sigma-Aldrich). After 24 hr, the medium was completely replaced by RPMI/B27 supplemented with 5 µM IWR-1 (Sigma-Aldrich) and 50 µg/mL ascorbic acid (Sigma-Aldrich). At day 3, i.e. 72 hr after day 0, medium was exchanged for RPMI/B27 supplemented with 5 µM IWR-1 until day 6. Medium was then exchanged every two days, until day 15. At day 15, cell preparations containing > 80% cardiac troponin T-positive CMs (confirmed by flow cytometry) were obtained.

### hPSC-CM culture

After a differentiation period of 15 days, hPSC-CMs were cultured for additional 20 days in four different culture media as indicated in Fig. 1A: GLCM: standard hPSC-CM medium (RPMI + B27); FAM: RPMI without Glc supplemented with B27, 1 mM glutamine (Gln), 100 µM oleic acid (OA)^20^, 50 µM palmitic acid (PA)^20^; GFAM: RPMI without Glc supplemented with B27, 1 mM Gln, 10 mM galactose (Gal)^13, 20^, 100 µM OA and 50 µM PA; and LACM&GFAM: 10 days in RPMI without Glc supplemented with B27, 1 mM Gln and 4 mM sodium L-Lactate (Sigma-Aldrich)^21^ and 10 days in GFAM. At days 10 (d10) and 20 (d20), hPSC-CMs were harvested for metabolic, transcriptomic, structural and functional analyses (more than 10 different batches were analyzed). hESC-CMs were harvested for metabolic and structural analyses (at least 3 different batches were analyzed).

### Metabolic and fluxomic analysis

#### Metabolite analysis

Glutamine (Gln), glutamate (Glu), glucose (Glc) and lactate (Lac) concentrations from cell culture supernatants were measured using YSI 7100MBS (YSI Incorporated, USA). Ammonia was quantified with an enzymatic assay kit (NZYTech, Portugal). Amino acid concentration was determined by HPLC using the Waters AcQ.Tag Amino Acid Analysis Method (Waters, Milford, MA), as described elsewhere^23^. The specific metabolic rates (qMet, expressed in nmol/(10^6^cell.h)) were calculated using the equation: qMet = ΔMet/(Δt × Xv), where ΔMet (mol/L) is the variation of metabolite concentration during the time period Δt (h), and Xv (cell/L) the average of cell concentration during the same time period.

#### Measurement of Oxygen Consumption Rate and Extracellular Acidification Rate

OXPHOS and glycolysis in hiPSC-CMs were estimated by measuring oxygen consumption rate (OCR) and extracellular acidification rate (ECAR) in real-time using an XF-96 Extracellular Flux Analyzer (Seahorse Biosciences) according to the manufacturer’s protocols. OXPHOS and glycolysis in hESC-CMs were determined using, respectively, the MitoXpress® Xtra - Oxygen Consumption Assay and the pH-Xtra™ Glycolysis Assay (Luxcel Biosciences Ltd.) following manufacturer’s recommendation. These fluorescence-based assays enable the direct, real-time, kinetic analysis of oxygen consumption rate and extracellular acidification rates^24^. Briefly, cells previously plated at approximately 70 000 cell/well in 96 well plates were treated with MitoXpress™ and pH-Xtra™ fluorescent reagents (separately) and fluorescence was monitored at Ex. 380 nm and Em. 670 (for MitoXpress™) and at Ex. 380 nm and Em. 615 (for pH-Xtra™), every 1 minute for 2 hours at 37 °C using a microwell plate fluorescence reader Spark 10 M (Tecan). Oxygen consumption rates (OCR) and extracellular acidification rates (ECAR) were calculated by measuring the slopes of the linear regions of florescence intensity curves using GraphPad Prism 6 software.

#### Isotopic tracer experiments and GC-MS analysis

For the parallel isotopic labelling experiments the following isotopic tracers were used: [1,2-^13^C]Glucose; [U-^13^C]Glutamine; [3-^13^C]Lactate; [U-^13^C]Galactose (all from Cambridge Isotope Laboratories) and [18-^13^C]Oleic Acid; [16-^13^C]Palmitic Acid (from Sigma-Aldrich). Only one isotopic tracer was used at a time to allow monitoring of the incorporation of each tracer. Label incorporation was characterized 24 and 48 h after label addition. Metabolic quenching, metabolite extraction and derivatization protocols were performed as described in the literature^25, 26^.

Metabolites were analyzed in a QP2010 mass spectrometer (Shimadzu) in the EI mode (70 eV) with a HP-5 MS column (Agilent Technologies). The obtained spectra were analyzed and integrated using GC-MS Solution software version 2.50 SU1 (Shimadzu). Mass Isotopomer Distributions (MID) were calculated after spectra integration and corrected for natural isotope abundance. The percentage of incorporation of each substrate in the TCA cycle was determined as the percentage of incorporation of each labelled substrate in TCA metabolites (citrate, fumarate and malate) normalized by the total incorporation provided by the main substrates present in the media in the same metabolites.

#### Metabolic network and nonstationary ^13^C-MFA

Nonstationary ^13^C-MFA of parallel labelling experiments was performed using the MATLAB-based software package INCA^27^, which automatically generates and simulates material balance equations from a user-defined metabolic network structure and experimental datasets. At least 10 restarts with random initial values for the parameters (intracellular fluxes and pools) were performed to find a global optimum. At convergence, the goodness-of-fit of the obtained solution was evaluated with a qui-square statistical test. The parameter continuation method was used to calculate 95% confidence intervals of the estimated fluxes^28^.

#### ATP production

ATP production was estimated using the reference value of ATP generated in glycolysis, TCA and β-oxidation^29^, multiplied by respective flux values determined in nonstationary ^13^C-MFA.

### Transcriptomic analysis

#### RNA Extraction, Amplification, Labeling, and BeadChip Hybridization of RNA Samples

Total RNA from hiPSC-CMs was extracted using the RNeasy Mini Kit (Qiagen). RNA quality was characterized by the quotient of the 28 S to 18 S ribosomal RNA electropherogram peak using an Agilent 2100 bioanalyzer and the RNA Nano Chip (Agilent). Transcriptional profiling was assessed using Illumina HumanHT-12 v4 Expression BeadChip microarray technology (Illumina) and data were processed and normalized as previously described^30^.﻿ Briefly, data were processed using the Environment for Statistical Computing 2.7.0 (R Foundation for Statistical Computing, Vienna, Austria, http://www.r-project.org) in combination with Bioconductor 2.2. The Bioconductor lumi package was used for quality control. Raw data were log2-transformed using the lumiT() function, and no background correction was performed^31^. Data were transformed using variance-stabilizing transformation and quantile normalized. All methods used were implemented in the R package lumi. Genes differentially expressed/enriched were identified using Student’s t test (p value < 0.05) and fold change FC ≥ |1.3|. 2D-Principal component analysis (2D-PCA), Euclidean distances between PCA centroids, Pearson correlation matrixes and density plots were generated using R version 3.3.2. PCA centroids for each group were defined as the center of gravity in the multidimensional space. The Euclidean distances between PCA centroids were calculated in the space defined by the first (PC1) and second (PC2) components. To determine the confidence interval around Pearson’ correlation coefficients Fisher’s r-to-z transformation was applied, then the limits of the confidence intervals were computed on the transformed scale and finally, the lower and upper limits of the coefficient interval were transformed back to the original scale, as described elsewhere^32, 33^. Kolmogorov-Smirnov test was used to calculate p-values in density Plots. Pathway analysis was performed using Ingenuity Pathway Analysis (Ingenuity Systems; Qiagen). For GO term analysis, GO term database (http://geneontology.org/) was used. Human left and right ventricular RNA samples, from a commercial supplier (Biocat #R1234138‐50‐BC and #R1234139‐50‐BC, respectively), were included in the analysis as positive control.

### Characterization of hPSC-CMs

#### Cell concentration and viability

Cell concentration and viability were determined by cell counting in a Fuchs–Rosenthal haemocytometer (Brand, Wertheim, Germany) using the trypan blue (Sigma-Aldrich) exclusion method (0.1% (v/v) in DPBS).

#### Transmission electron microscopy (TEM)

Monolayers of human hiPSC-CMs were fixed in 2% (v/v) glutaraldehyde in 0.1 M HEPES buffer (pH 7.4) for 20 min, washed in DPBS and stored in 1% (v/v) glutaraldehyde in DPBS until analyses. Samples were washed, contrasted, dehydrated, infiltrated, embedded, cured, dissected into small blocks and mounted on epon dummy blocks, as previously described^34^. Ultrathin sections of cell monolayers were cut on a Leica UC6 ultramicrotome using a diamond knife. Sections were collected on formvar-coated slot grids, stained with lead citrate and uranyl acetate as described in the literature^35^, and analyzed on a FEI Morgagni 268 at 80 kV. Images were taken with an Olympus MegaView III using the iTEM software.

#### Sarcomere Alignment

Sarcomere alignment was determined as previously described^7^. Briefly, alignment is defined by the inverse of the magnitude of angle dispersion (the standard deviation of angles of sarcomere edges measured in ImageJ software (National Institutes of Health)). Low angle dispersion indicates high degree of alignment. Rose plot was generated using Mathematica (Wolfram Research Inc.).

#### Immunofluorescence microscopy

Cells were washed with DPBS and fixed in 4% (w/v) Paraformaldehyde (PFA) in DPBS solution for 15 min. After being permeabilized and blocked with 5% (v/v) FBS, 1% (w/v) BSA, 0.3% (v/v) Triton X-100 in DPBS for 1 h, at room temperature (20–25 °C), cells were incubated with primary antibodies diluted in 1% (w/v) BSA, 0.1% (w/v) TX-100 in PBS overnight at 4 °C. The following primary antibodies were used: α-sarcomeric actinin (1:200, Sigma-Aldrich), titin (1:100, Santa Cruz Biotechnology) and troponin I (1:100, Millipore). Afterwards, cells were washed in DPBS and incubated with secondary antibody, Alexa Fluor 594 goat anti-mouse IgG (1:500, Invitrogen), in the dark for 1 h at room temperature (20–25 °C). Cell nuclei were counterstained with Hoechst 33342 nucleic acid dye (1:1000, Thermo Scientific). Representative images were taken using an inverted fluorescence microscope (DMI 6000, Leica).

#### Cell morphology

Cell area, circularity index and aspect ratio were determined in ImageJ software using standard analysis plugins. For each condition, at least 70 cells were analyzed.

#### Measurement of mitochondrial membrane potential

Differences in membrane potential, were assessed using MitoView™ 633 (Biotium, Inc., California, USA) following manufacturer’s instructions. Briefly, after incubation with the dye for 1 h, fluorescence was measured in a microwell plate fluorescence reader, Infinite 200 PRO NanoQuant (TECAN).

#### Lipid droplets content

Lipid droplet content was assessed using the Lipid Droplets Fluorescence Assay Kit (Cayman, USA), according to the manufacturer’s instructions. Fluorescence was quantified in a microwell plate reader, Infinite 200 PRO NanoQuant (TECAN). Representative images were taken using an inverted fluorescence microscope (DMI 6000, Leica).

### Assessment of hiPSC-CM functionality

#### Dissociation and Plating

hiPSC-CMs were dissociated into single-cell suspensions by incubation with TrypLE^TM^Select for 5 min. Single hiPSC-CM were seeded on Matrigel-coated Fluorodishes at 2,000–6,000 cells/cm^2^. Single contracting hiPSC-CM and CM clusters (<10 cells) growing on PDMS substrates for 3–5 days were chosen for image acquisition.

#### Confocal Imaging

hiPSC-CMs were imaged in the different culture media inside a climate control chamber at 37 °C with 5% CO_2_ (Pathology Devices) using a Nikon A1R confocal microscope with DIC microscopy using a 488- or 647-nm laser and an anti-vibration table (TMC). Movies were acquired with at least 100 fps. Movies of CMs displaying at least five contraction cycles were recorded at baseline and then 5 min after each treatment. Movies were acquired without and with field-stimulated with 10 V at 1 Hz using the RC- 37WS perfusion insert with electrodes (Warner Instruments) connected to a C-Pace EP stimulator (Ion Optix).

#### Calcium and Action Potential imaging

For calcium imaging, 0.5 mM of Fluo-4 AM (Life Technologies) was added to the CM culture medium 10 min prior to image acquisition. For action potential imaging, FluoVolt membrane potential kit (Life Technologies) was used at a dilution of 1:1000 according to the manufacturer’s protocol. Both fluorescence and DIC channels were recorded concurrently for multiple contraction cycles.

#### Data Analysis and Display

The Nikon Elements software package and Fiji were used for movie file conversions^36^. Movies were cropped using Fiji to isolate the contracting CMs from neighboring cells and cellular debris. Contractile kinetics and force generation by the CMs were analyzed using the method developed by Kijlstra *et al*.^37^. Specifically, movies were loaded into Visible™ software (Reify) to generate a similarity matrix of n rows by n columns, where n is the number of frames in the movie. Analysis of the similarity matrix was performed in Excel (Microsoft). For analysis of contraction curves, at least five contraction curves from a single video were averaged by matching the start of the contractions. Curve-fitting was applied to this averaged curve to obtain accurate measurements of maximum shortening and relengthening velocities.

#### Contractile Force Determination

Contractile forces generated by shortening CMs were calculated in MATLAB based on the contractility curve generated with BASiC^37^, the dimensions of the CMs, and the mechanical properties of the PDMS substrate^38^. Variable input for the MATLAB script is the width of the cell in centimeters, the length of the cell (in the direction of contraction) in centimeters, and the amount of cell shortening in centimeters. The Young’s modulus of PDMS substrates was 5 kPa for all experiments.

#### Statistical analysis

Statistical analysis was performed using GraphPad Prism Software version 5. Values are represented as mean ± SD or as mean ± SEM of independent measurements or assays (at least n = 3 replicates were considered). Statistical significance was evaluated using either unpaired Student’s t test or one-way analysis of variance (ANOVA). Values of P < 0.05 were considered statistically significant.

## Results

### hiPSC-CMs shift their metabolism from glycolysis to OXPHOS in the absence of glucose

Beating hiPSC-CMs generated after 15 days of differentiation period, exhibit a typical fetal-like glycolytic metabolism, as confirmed by a high Lac production (Fig. 1B), high qLac/qGlc ratio (Fig. 1C), and a high fraction of Lac (90%) derived from [1,2-^13^C]Glc upon 48 h incubation (Fig. 1D). These metabolic indicators as well as the fraction of intracellular TCA cycle pools derived from [1,2-^13^C]Glc and [U-^13^C]Gln, do not change significantly when keeping hiPSC-CMs in Glc rich medium (GLCM) during 20 days (Fig. 1D,E), reflecting no improvements in the oxidative capacity of the cells when cultured in GLCM. This metabolic picture changes when removing Glc from the medium and adding FA alone (FAM) or together with Gal (GFAM), or Lac in the first 10 days followed by GFAM (LACM&GFAM) (Fig. 1B). hiPSC-CMs were able to consume all new substrates and stop Lac secretion (Fig. 1B) showing remarkable metabolic plasticity. In both GFAM and LACM&GFAM, hiPSC-CMs displayed a significantly higher oxygen consumption rate (OCR, Fig. 1F), lower extracellular acidification rate (ECAR, Fig. 1G) and consequently higher OCR/ECAR ratio (Fig. 1H), reflecting the higher contribution of OXPHOS comparatively to glycolysis for energy generation in these conditions.

To further investigate the changes in central carbon metabolism, we cultured hiPSC-CMs in the conditions described above using uniformly ^13^C-labelled versions of the corresponding nutrients and analyzed the incorporation of ^13^C into TCA cycle intermediates (citrate, fumarate and malate). In FAM, at day 0, 18% of the labelled fraction of these metabolites came from [U-^13^C]PA and [U-^13^C]OA and the remaining 82% from [U-^13^C]Gln (Fig. 1I), suggesting that in FAM glutaminolysis is an important pathway to support TCA cycle anaplerosis when compared with GLCM, where just a small portion of the TCA cycle pools incorporates carbon from [U-^13^C]Gln (Fig. 1E). In the presence of Gal (GFAM), the incorporation of Gln decreased to 38%, whereas [U-^13^C]Gal accounted for approximately 40% of the label in TCA cycle (Fig. 1J). Notably, after 10 days in FAM the contribution from FA to TCA cycle label increased to 35% (Fig. 1I). A similar adaptation of hiPSC-CMs to FA consumption was observed in GFAM, in which FA contribution to labelled TCA cycle pools increased from 19% (day 0) to 36% (day 20) (Fig. 1J). For hiPSC-CMs cultured in LACM&GFAM, the incorporation of FA in TCA intermediates was similar at days 10 and 20 (approximately 30%, Fig. 1K), suggesting that initial reliance in Lac oxidation may precondition hiPSC-CMs, allowing for a better adaptation to FA metabolization upon initial exposure to this substrate. Also, like in GFAM, there is a decrease in Gln contribution concomitant with increased incorporation of Gal (Fig. 1J,K). Overall, these results highlight the metabolic plasticity of the hiPSC-CMs in the absence of Glc and in specific the increased role of FA and Gal as fuel of TCA cycle during culture in FAM and GFAM in detriment of Gln.

### Culture of hiPSC-CMs in fatty acid medium lacking sugar source induces lipotoxicity

**Figure 2:**
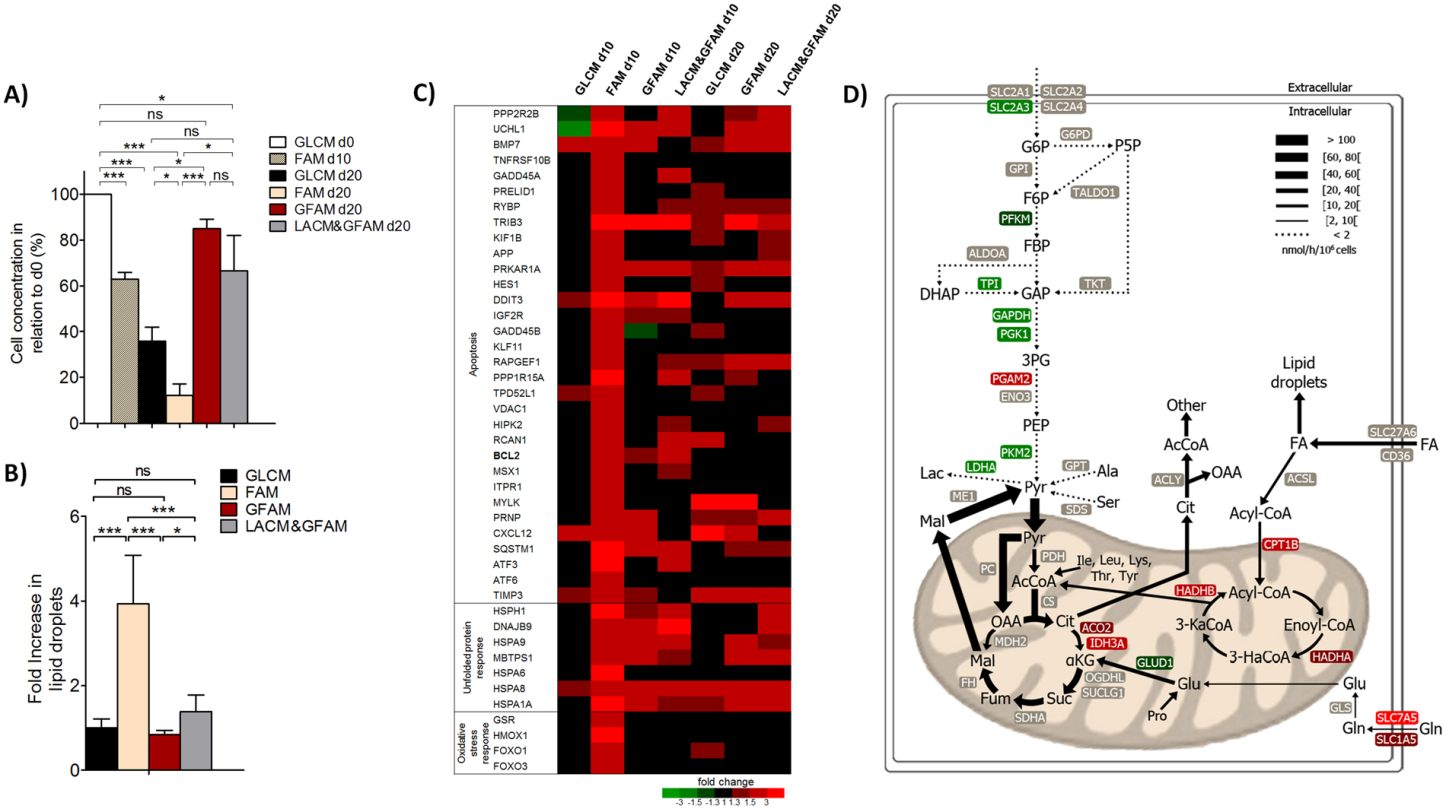
In the absence of a sugar source, fatty acid rich medium induces lipotoxicity in hiPSC-CMs. (**A**) Percentage of cell concentration, in all conditions tested, in relation to day 0 (d0), assessed using the Trypan Blue Exclusion Method. (**B**) Analysis of lipid droplet content using a lipid droplet-specific fluorescent dye (Nile Red) of hiPSC-CMs at day 15. Fluorescence values were normalized for the values obtained in cells cultivated in GLCM. Data are represented as mean ± SD. *p < 0.05; ***p < 0.001; ns, not significant. n ≥ 10 separate experiments. (**C**) Heat map of differentially expressed genes closely related with apoptosis, unfolded protein and oxidative stress responses in hiPSC-CMs cultivated in different culture media. Genes were considered up- or down-regulated based on a fold change in expression FC ≥ |1.3| compared to d0. Heatmap shows averaged values from n = 2. (**D**) Metabolic flux map of the central carbon metabolism of hiPSC-CMs cultivated for 10 days in FAM. The flux map was determined using non-stationary ^13^C-MFA. Arrow thickness reflects flux values (see Table S1 for exact flux values).

Our results show that when cultured in FAM, hiPSC-CMs exhibited: i) a drastic decrease in cell concentration from day 10 onwards, reaching about 10% of the initial cell concentration at day 20 (Fig. 2A) and ii) a significant 4-fold increase in lipid droplet content when compared with hiPSC-CMs cultured in other media (Fig. 2B). These results suggest that FA supply has surpassed the cellular oxidative capacity, leading to the intracellular accumulation of FA that has probably caused cellular damage and toxicity^39^. Several studies have shown that cell exposure to high FA levels can affect multiple cellular processes such as reactive oxygen species (ROS) production, causing mitochondria, endoplasmic reticulum and lysosomal dysfunction and ultimately apoptosis^40^. Whole-genome transcriptome analysis revealed that FAM induced up-regulation of apoptosis-related genes and genes that encode for mediators of oxidative stress resistance and heat shock proteins (Fig. 2C). The activation of these genes reflects an adaptive mechanism of the cells to hamper cardiac damage associated with high FA exposure. To further characterize the global changes in central carbon metabolism of hiPSC-CMs cultured in FAM when compared to other feeding strategies, uptake and production rates were integrated with the ^13^C labelling profiles of intracellular metabolites from multiple tracers in a metabolic network model using non-stationary metabolic flux analysis (MFA)^41^. Reasonable fits were obtained (Figs S1–S3) and the estimated fluxes and associated 95% confidence intervals are provided in Table S1. The flux map of hiPSC-CMs cultured in FAM at day 10, confirms that most of the consumed FA (74%) is channeled to internal stores (lipid droplets) instead of entering β-oxidation (Fig. 2D). Therefore, even though FA are the main substrate of the adult heart^16^, prolonged culture of hiPSC-CMs in FA-rich medium lacking sugar source lead to FA accumulation and alterations in the central carbon metabolism which could justify the decrease in cell viability.

### Galactose prevents lipotoxicity in hiPSC-CMs cultured in fatty acid medium and improves cellular oxidative capacity

**Figure 3:**
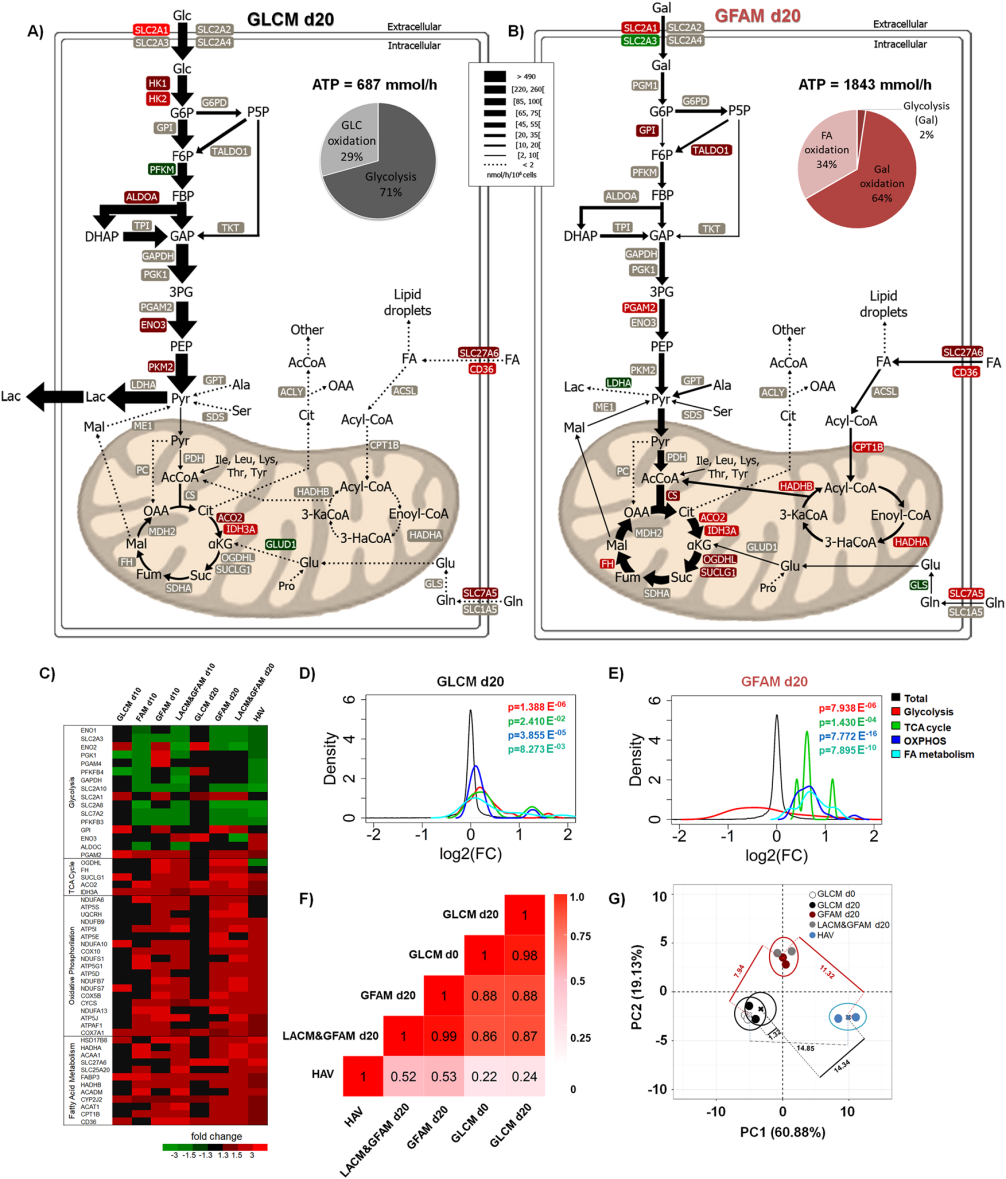
Effect of culture media composition on the fluxome and metabolic transcriptome of hiPSC-CMs. Metabolic flux maps highlighting central carbon metabolism of hiPSC-CMs cultured in GLCM (**A**) and GFAM (**B**). The contribution of each metabolic pathway for ATP production is specified in the pie chart and the total amount of ATP produced is indicated above the pie chart. Up-regulated and down-regulated genes encoding metabolic enzymes are demarked in red and green, respectively. (**C**) Heat map of differentially expressed genes associated with metabolic processes/pathways. A complete list of the genes and respective FC can be found in Table S2. Heatmap shows averaged values from n = 2. (**D**–**E**) Density plots generated with fold change expression of genes from the four metabolic processes showed in (**C**), for hiPSC-CMs in GLCM (**D**) and GFAM (**E**) at day 20. X axis indicates log_2_ fold change in gene expression. Black line indicates expression of all genes. Colored lines toward the left and right side of the black line indicate down-regulation and up-regulation of pathways, respectively. Kolmogorov-Smirnov test was used to calculate p-values. (**F**) Pairwise Pearson correlation coefficients using expression data for the metabolic processes showed in (**C**). The highest and the lowest coefficients are colored in a red to white gradient. (**G**) 2D principal component analysis using expression data for the metabolic processes showed in (**C**). Euclidean distances between PCA centroids of each condition, calculated considering the first (PC1) and second (PC2) components, are also highlighted.

When cultured in GFAM, hiPSC-CMs showed unaltered cell concentration throughout culture time (Fig. 2A) and a significant lower intracellular FA accumulation than in FAM (Fig. 2B), suggesting that combining FA with Gal supplementation constitutes a better approach to culture hiPSC-CMs avoiding lipotoxicity. A comparison of the flux maps of hiPSC-CMs in GLCM and GFAM at day 20, reveals that hiPSC-CMs maintained in GFAM exhibited higher oxidative metabolism as indicated by a lower flux through glycolysis (around 10-fold) and higher flux through TCA cycle (over 6-fold) (Fig. 3A,B). Most of the cytosolic Pyr was transported to the mitochondria and converted to AcCoA through pyruvate dehydrogenase (PDH) activity (72.4 in GFAM vs 5.6 nmol/(10^6^cells.h) in GLCM), whereas in GLCM most of the cytosolic Pyr was diverted to Lac secretion (491.4 in GLCM vs 0.0 nmol/(10^6^cells.h) in GFAM), consistent with the measured extracellular rates (Fig. 1B). A pronounced effect of GFAM on hiPSC-CMs central carbon metabolism was the up-regulation of the β-oxidation pathway, achieving fluxes of 11 nmol/(10^6^cells.h) whereas in GLCM these fluxes were negligible, underscoring the increased FA oxidation in GFAM (Fig. 3A,B). Importantly, hiPSC-CMs cultured in LACM&GFAM and GFAM showed similar fluxomes (data not shown).

The estimated changes in metabolic fluxes correlate with alterations in the metabolic transcriptome. Specifically, we observed a significant down-regulation of glycolytic genes and a significant up-regulation of most genes related to TCA cycle, OXPHOS and FA metabolism in GFAM and LACM&GFAM cultures, a trend similar to that seen in human adult ventricular (HAV) tissue (Figs 3C–E and S4). Using gene expression data from these four metabolic pathways, we carried out a Pearson correlation analysis and a 2D principal component analysis (2D-PCA) comparing hiPSC-CMs at day 0 and after 20 days of culture in GLCM, GFAM, and LACM&GFAM with HAV tissue. Pearson correlation coefficients revealed that GFAM (0.53; 95% CI: 0.51–0.55) and LACM&GFAM (0.52; 95% CI: 0.50–0.55)) present a significantly higher correlation with HAV than GLCM cultures (0.24; 95% CI: 0.21–0.27); Fig. 3F and Table S3). Additionally, 2D-PCA analysis clustered together hiPSC-CMs in GLCM at day 0 and at day 20, separating these samples from HAV in principal component 1 (PC1, 60.88% variance, Fig. 3G), while hiPSC-CMs cultured in GFAM and LACM&GFAM at day 20 were placed in the middle of PC1 and closer to HAV tissue (Fig. 3G). Euclidean distances calculated considering both PC1 and PC2 (80.01% variance, Table S4) corroborated these results showing that GFAM (11.08 ± 0.94) and LACM&GFAM (11.55 ± 2.50) are closer to HAV tissue than GLCM (14.34 ± 1.08). Together, these data re-enforce the idea that hiPSC-CMs cultured in GFAM and LACM&GFAM show a metabolic transcriptome more similar to HAV tissue.

We noticed a faster down-regulation of glycolysis related genes (such as ALDOC; GAPDH; PGK1) in LACM&GFAM than in GFAM (Fig. 3C). This indicates that the first 10 days of culture in LACM contributes for a sharp inactivation of the glycolytic pathway whereas the presence of Gal in GFAM lead to a more gradual decrease in the expression of glycolytic genes. Of particular interest is the up-regulation of enzymes glucose-6-phosphate isomerase (GPI) from glycolysis and transaldolase (TALDO1) from the non-oxidative branch of pentose phosphate pathway (PPP) in GFAM (Fig. 3B,C). Gal is converted into G6P inside the cells, and the G6P formed can enter either the glycolytic pathway or the PPP. Thus, the increased expression of GPI and TALDO1 may reflect an adaptive response of the cells to enhance Gal metabolization. Interestingly, the PPP flux was similar in GFAM and GLCM, but in GFAM represented approximately 87% of the respective glycolytic fluxes and in GLCM just 13% (Fig. 3A,B).

The increased expression of genes related with FA metabolism (CD36, CPT1B, SLC25A20, HADHA/B) (Fig. 3C) suggests that hiPSC-CMs in GFAM and in LACM&GFAM adapt also at the transcriptional level to more efficiently consume FA. Also, the higher expression of OXPHOS related genes (specifically, ATP5, COX and NDUF family of genes) (Figs 3C and S5), re-enforces the idea that mitochondrial oxidative metabolism is enhanced in both media. In accordance, the estimated ATP production increased 2.7-fold in GFAM (1843 nmol/h) comparing with GLCM (687 nmol/h) (Fig. 3A,B). Taking into account the estimated fluxes and the reference amount of ATP produced *per* each substrate molecule^42^, glycolysis accounts for 71% of the total produced ATP in GLCM, whereas in GFAM, glycolysis originates just 2% of the total ATP. In GFAM the majority of the ATP (98%) is produced through oxidation of Gal (64%) and FA (34%) (Fig. 3A,B, pie charts).

The lower Gln consumption rates in GLCM and GFAM (1.0 and 2.6 nmol/(10^6^cells.h), respectively), suggests an almost negligible role of Gln in the metabolism of hiPSC-CMs cultured in these media.

Overall, ^13^C-MFA and transcriptome analysis confirmed that in GLCM the majority of Glc is metabolized by glycolysis originating Lac, whereas in GFAM, FA and Gal are both oxidatively metabolized via TCA cycle and OXPHOS for ATP generation, providing additional evidence that hiPSC-CMs switch their metabolism from a fetal-like glycolytic metabolism to a more energetically efficient adult-like oxidative metabolism when cultured in GFAM (and LACM&GFAM).

### Adaptation to LACM induces higher cell death than GFAM

The up-regulation of some genes related with unfolded protein response in both LACM and GFAM at day 10 (Fig. 2C), suggests that Glc depletion *per se* induced a stress for the cells resulting in the activation of survival signaling cascades. Nonetheless, the cell death (Figs 2A and S6A) and the up-regulation of apoptotic genes (Fig. 2C) were higher in hiPSC-CMs cultured in LACM, suggesting that the metabolic adaptation to Lac consumption is more harmful for the cells than the adaptation to Gal and FA consumption. It should be highlighted that the hiPSC-CMs used in this study were already non-proliferative, as verified by the absence of ki-67 expression at day 0 (Fig. S6B). The lack of significant enrichment in cell cycle related pathways, from day 0 to day 20, in all culture media (Fig. S6C), also suggest that culture in different media did not affect hiPSC-CM proliferative capacity.

### hiPSC-CMs cultured in GFAM or LACM&GFAM present transcriptional signatures closer to human ventricular CMs

**Figure 4:**
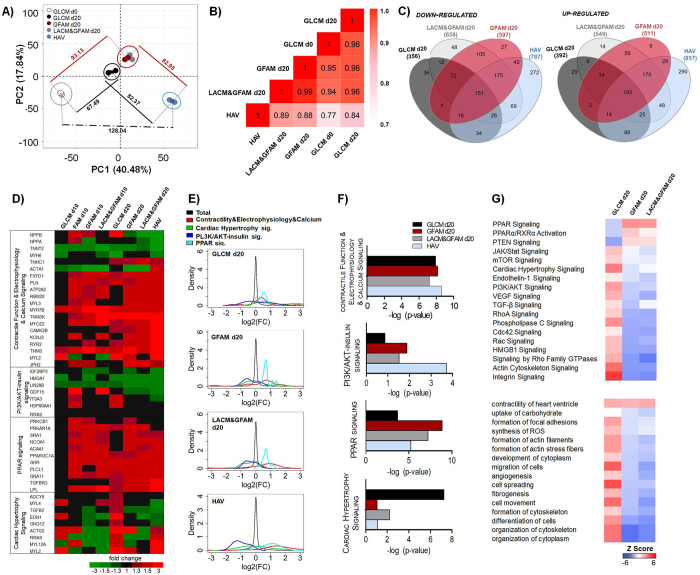
Effect of culture medium composition on hiPSC-CM transcriptional profiling. (**A**) 2D principal component analysis of all genes significantly enriched (p < 0.01) in hiPSC-CM cultures at day 0, after 20 days of culture in GLCM, GFAM and LACM&GFAM and HAV tissue samples. Euclidean distances between PCA centroids of each condition, calculated considering PC1 and PC2, are also highlighted. (**B**) Pairwise Pearson correlation coefficients using expression data of all enriched genes (p < 0.01). (**C**) Venn diagrams of overlapping differentially-expressed genes (p < 0.01 and FC ≥ |1.3|) among GLCM, GFAM, LACM&GFAM cultures at day 20 and HAV tissue. (**D**) Heatmap depicting changes in the expression of genes involved in biological processes/pathways closely associated with CM development and function. (**E**–**F**) Density plots generated with fold change expression of genes from the four cardiac-related categories shown in (**D**), for day 20 cultures (GLCM, GFAM and LACM&GFAM). Black line indicates expression of all genes. Colored lines toward the left and right side of the black line indicate down-regulation and up-regulation of pathways, respectively. Kolmogorov-Smirnov test was used to calculate p-values. (**G**) Heatmaps highlighting the activation status of canonical pathways (upper panel) and biological functions (lower panel) predicted by IPA. Functions/pathways were considered significantly activated (or inhibited) with an overlap p-value ≤ 0.05 and an IPA activation Z-score ≥ |2.0|. Red indicates positive Z-score (activated function) and blue indicates negative Z-score (inhibited function).

Whole transcriptome analysis showed that global gene expression patterns of hiPSC-CMs change gradually and differently along culture time in distinct media. 2D-PCA of all differently expressed genes (p-value < 0.01 between the analyzed sample groups) clearly separated HAV the farthest from hiPSC-CMs at day 0 in PC1, that accounted for most of the data variance (40.48%; Fig. 4A). GLCM, GFAM and LACM&GFAM cultures were placed in the middle of the PC1 axis, but GLCM was positioned closer to day 0 and GFAM and LACM&GFAM closer to HAV, in PC1 (Fig. 4A). However, Euclidean distances from PCA centroids of GLCM, GFAM and LACM&GFAM to HAV, considering both PC1 and PC2, were not meaningfully different (Table S4). Nonetheless, gene expression of hiPSC-CMs maintained in GFAM and LACM&GFAM for 20 days showed significantly higher correlation with HAV (0.88; 95% CI: 0.87–0.88 and 0.89; 95% CI: 0.88–0.89, respectively) than hiPSC-CMs in GLCM with HAV (0.84; 95% CI: 0.83–0.85) (Fig. 4B and Table S5). In agreement, Venn diagrams showed that both GFAM and LACM&GFAM present more overlapping of significantly enriched genes with HAV tissue than GLCM (Fig. 4C). Although, some results may suggest that transcriptional signatures of hiPSC-CMs cultured in GFAM and LACM&GFAM were more similar to that of HAV tissue, used as reference of mature CMs, than of hiPSC-CMs cultured in GLCM, the proximity of GFAM and LACM&GFAM treated hiPSC-CMs to adult tissue is not so notary, as expected, than when looked only at metabolic transcriptome.

### GFAM and LACM & GFAM enhance the expression of cardiac markers

When compared with hiPSC-CMs at day 0, hiPSC-CMs maintained in GFAM and LACM&GFAM for 20 days, exhibit enhanced expression of cardiac genes related with i) contractile function and sarcomere structure (e.g. MYL3, TNNC1, TNNI3), ii) calcium cycling (e.g. ATP2A2 and RYR2), and iii) electrophysiological properties (e.g. KCNJ2) (Fig. 4D). Of particular interest is the 2-fold up-regulation of KCNJ2, gene that suffices to render the electrophysiological phenotype indistinguishable from the adult counterparts^11^.

The expression of genes typically enhanced in fetal-like CMs and repressed in adult ventricular CMs (e.g. NPPB and ACTA1)^2^, decreased in hiPSC-CMs cultured in GFAM and LACM&GFAM. In hiPSC-CMs cultured in GLCM, the expression of NPPB and ACT1, was reactivated and kept similar to those of the immature hiPSC-CMs (day0), respectively. Although ACTA1 is part of the fetal gene program in CMs, this gene is also expressed by other cardiac cell types, including smooth muscle cells, which may justify its up-regulation in HAV tissue. Notably, expression of the TNNI3 gene that encodes for the adult cardiac troponin I (cTnI), a key hallmark of human CM maturation *in vivo* ^43^, was up-regulated in all culture conditions tested, however this increase was higher in GFAM (Fig. 4D). It should be noted that up-regulation of genes related with contractile function, calcium signaling and electrophysiology was observed already at day 10 in GFAM whereas in GLCM, only minor improvements were observed at day 20 (Fig. 4D). Additionally, a more in-depth evaluation of the data using density plots revealed that in GFAM cultures the expression of these genes follows a distribution more similar to that observed in HAV tissue (Fig. 4E,F).

Examination of the transcript levels of all genes in the abovementioned samples, using IPA revealed the enrichment of several canonical pathways known to be critical for *in vivo* heart development. Inhibition of PI3K/AKT/insulin signaling and activation of PPAR signaling were consistently predicted by IPA and density plots in both LACM&GFAM and GFAM, whereas the opposite trend was observed in GLCM (Fig. 4E–G). PPARα is a major transcriptional regulator abundantly expressed in heart^44^. Specifically, PPARα regulates the expression of genes involved in FA uptake, cytosolic FA binding and esterification, FA β-oxidation, and Glc oxidation^44, 45^. PPARs activation have been related with the up-regulation of FA metabolism and with down-regulation of PI3/AKT/insulin pathway, which is involved in Glc metabolism regulation^46^. Of note is the up-regulation of PPARGC1A, observed already at day 10 in GFAM and LACM&GFAM (Fig. 4D), that encodes for PGC-1α, a transcriptional coactivator highly expressed in cardiac tissue^47^. Up-regulation of PGC-1α results in increased mitochondrial biogenesis, FA β-oxidation and OXPHOS^48, 49^, features already described as increased in GFAM and LACM&GFAM (Fig. 3). The inverse relationship between FA metabolism/PPARα signaling and PI3K/AKT/insulin signaling observed in GFAM and LACM&GFAM cultures is consistent with the metabolic switch detected in these cultures and is in agreement with the pattern reported for 1-year matured hESC-CMs^50^.

Of note is that both IPA and density plots revealed that the cardiac hypertrophy pathway is significantly up-regulated in GLCM cultures. Specifically, the main canonical pathways and biological functions, predicted by IPA, as activated in GLCM and inactivated in GFAM and LACM&GFAM were cardiac hypertrophy and related pathways/biological functions, including JAK/Stat, mTOR, Endothelin-1, Phospholipase C, TGF-β, actin cytoskeleton and integrin signaling, formation of actin stress fibers, angiogenesis and fibrogenesis (Fig. 4G)^51–53^.

### GFAM improves the structure and ultrastructure of hiPSC-CMs

**Figure 5:**
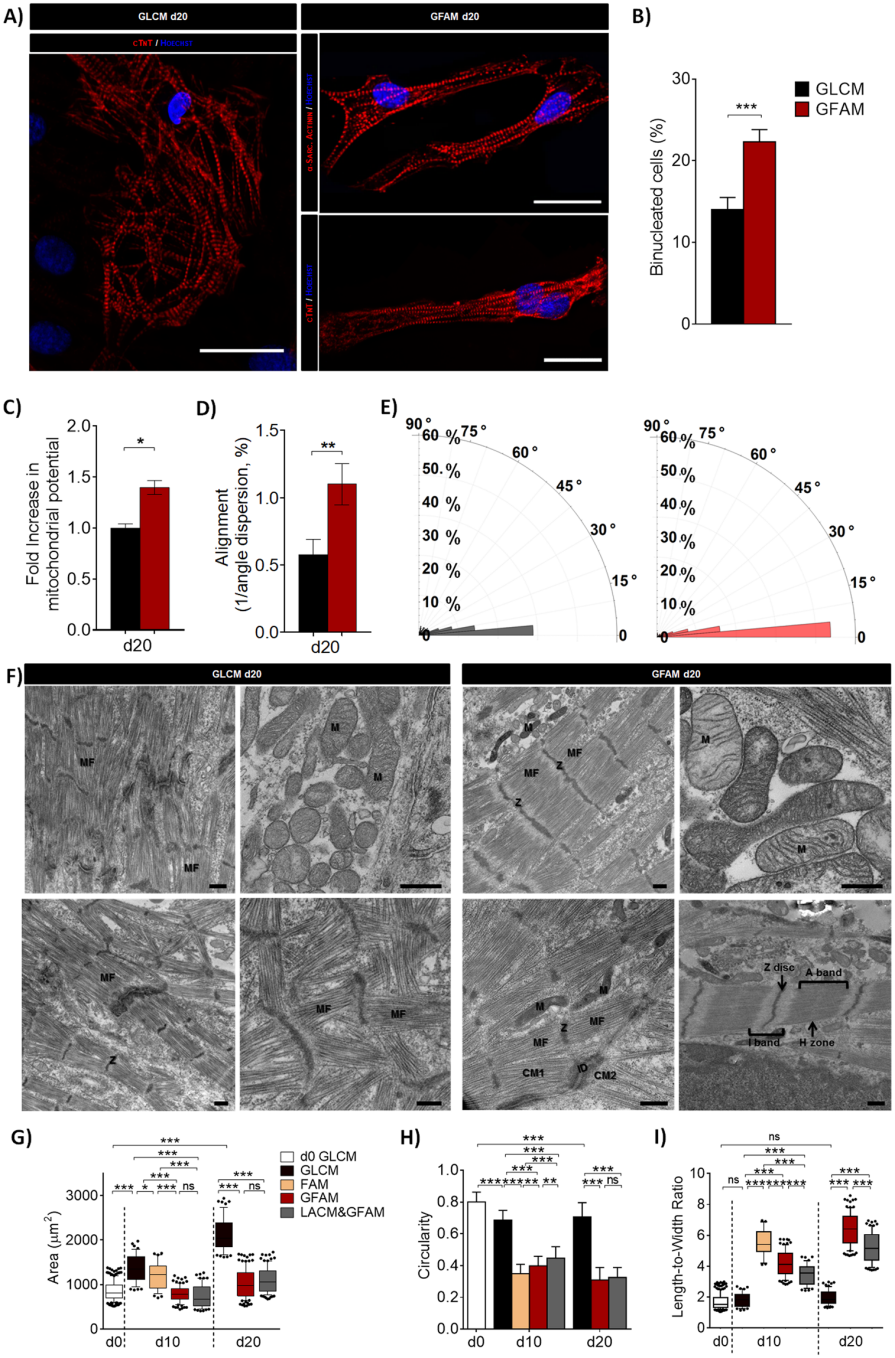
Structural and ultrastructural analyses of hiPSC-CMs after culture in different media. (**A**) Representative images of hPSC-CMs immunostained for cardiac troponin-T (cTnT) and α-sarcomeric actinin (red). Nuclei (blue) were stained with Hoechst 33342. Scale bars = 30 μm. (**B**) Percentage of binucleated hiPSC-CMs in GLCM and GFAM (n > 20 per condition). **C**) Fold increase in mitochondria potential assessed by the mitochondria specific dye Mitoview. Fluorescence values were normalized for the values obtained in cells cultivated in GLCM. (D-E) Sarcomere alignment in hiPSC-CMs cultured for 20 days in GLCM and GFAM: (**D**) Inverse of the magnitude of sarcomere angle dispersion and (**E**) Rose plot histogram of sarcomere orientation. n > 30 sarcomeres per condition. (**F**) TEM images of hiPSC-CMs. Myofibrils (MF), Z-disks (Z), sarcomeric bands: A- and I-bands with a H-zone, intercalated disks (ID) connecting adjacent CMs and Mitochondria (M) are highlighted. Scale bars = 500 nm. (**G**–**I**) Cell structure characterization in terms of cell area (**G**), circularity index (**H**), length-to-width ratio (**I**). n > 70 cells per condition from at least 5 separate experiments. Data are represented as mean ± SD. *p < 0.05; **p < 0.01; ***p < 0.001; ns, not significant.

Immunofluorescence and TEM analyses corroborated the gene expression data, confirming an improved structural maturation of hiPSC-CMs cultured in GFAM (and in LACM&GFAM) comparatively to GLCM. hiPSC-CMs cultured in GFAM exhibited: i) well defined cross-striated patterns of sarcomeric α-actinin and cTnT; ii) significantly higher (1.6-fold) percentage of binucleated cells; ii) high density of aligned myofibrils composed by sarcomeres with organized Z-disks, A- and I-bands and a distinctive H-zone; iii) significantly higher degree of sarcomere alignment; and iv) more mitochondria with prominent cristae close to the myofibrils (Fig. 5A–F). On the other hand, hiPSC-CMs maintained in GLCM showed disorganized and diffuse cTnT staining (Fig. 5A) and a poorly organized contractile machinery, characterized by low myofibril density and orientation, and variable Z-disc alignment (Fig. 5D–F). In accordance, Mitoview fluorescence (inner mitochondrial membrane potential dye), was significantly higher in GFAM in relation to GLCM conditions (Fig. 5C).

Moreover, significant changes in cell morphology were observed through time in all conditions (Fig. 5G–I). When compared to GLCM, hiPSC-CMs cultured in GFAM or LACM&GFAM exhibited significantly lower cell area (Fig. 5G) and circularity index (Fig. 5H) and higher length/width ratio (Fig. 5I). These numbers are similar to the values previously reported for adult human ventricular CMs^2^. For example, length/width ratios in the range of 5-9.5 have been reported for adult CMs^1^ and we observed an average ratio of 6.4 in hiPSC-CMs cultured in GFAM.

The significant increase in cell surface area and spherical morphology observed in hiPSC-CMs maintained in GLCM (Fig. 5G–I), reflects again the propensity of hiPSC-CMs to undergo hypertrophy.

### Effect of GFAM on CM metabolism and structure is cell line independent

CMs derived from an independent hPSC line (HES3-NKX2–5(eGFP/w)), designated hereafter as hESC-CMs, were also treated with GFAM for 20 days and compared with hESC-CMs maintained in GLCM for the same time period in terms of cellular metabolism and structure. hESC-CMs cultured GFAM, also displayed an over-reliance on oxidative phosphorylation relative to glycolysis (as demonstrated by i) a significant decrease in extracellular acidification rate (ECAR, Fig. S7A), ii) a significant increase in oxygen consumption rate (OCR, Fig. S7B) and iii) a significant OCR/ECAR ratio (Fig. S7C) relatively to hESC-CMs maintained in GLCM). Additionally, hESC-CMs cultured in GFAM showed a 1.6-fold increase in the percentage of binucleated cells when compared with GLCM cultures (Fig. S7D). Immunofluorescence microscopy analyses revealed that hESC-CMs cultured in GFAM also present organized sarcomeric structures and an elongated rod-shaped morphology (Fig. S7E) in contrast with hESC-CMs cultured in GLCM that display a rounded morphology with a disorganized cardiac structure (Fig. S7E). Detailed morphological analysis confirmed that GFAM treated cells present a significantly lower cell area (Fig. S7F) and circularity index (Fig. S7G) as well as significantly higher length/width ratio (Fig. S7H) compared with GLCM treated cells. All together these findings indicate that GFAM induce metabolic and structural maturation of CMs derived from different hPSC lines, confirming the robustness of this approach.

### GFAM improves calcium transients, contractility and action potential kinetics of hiPSC-CMs

**Figure 6:**
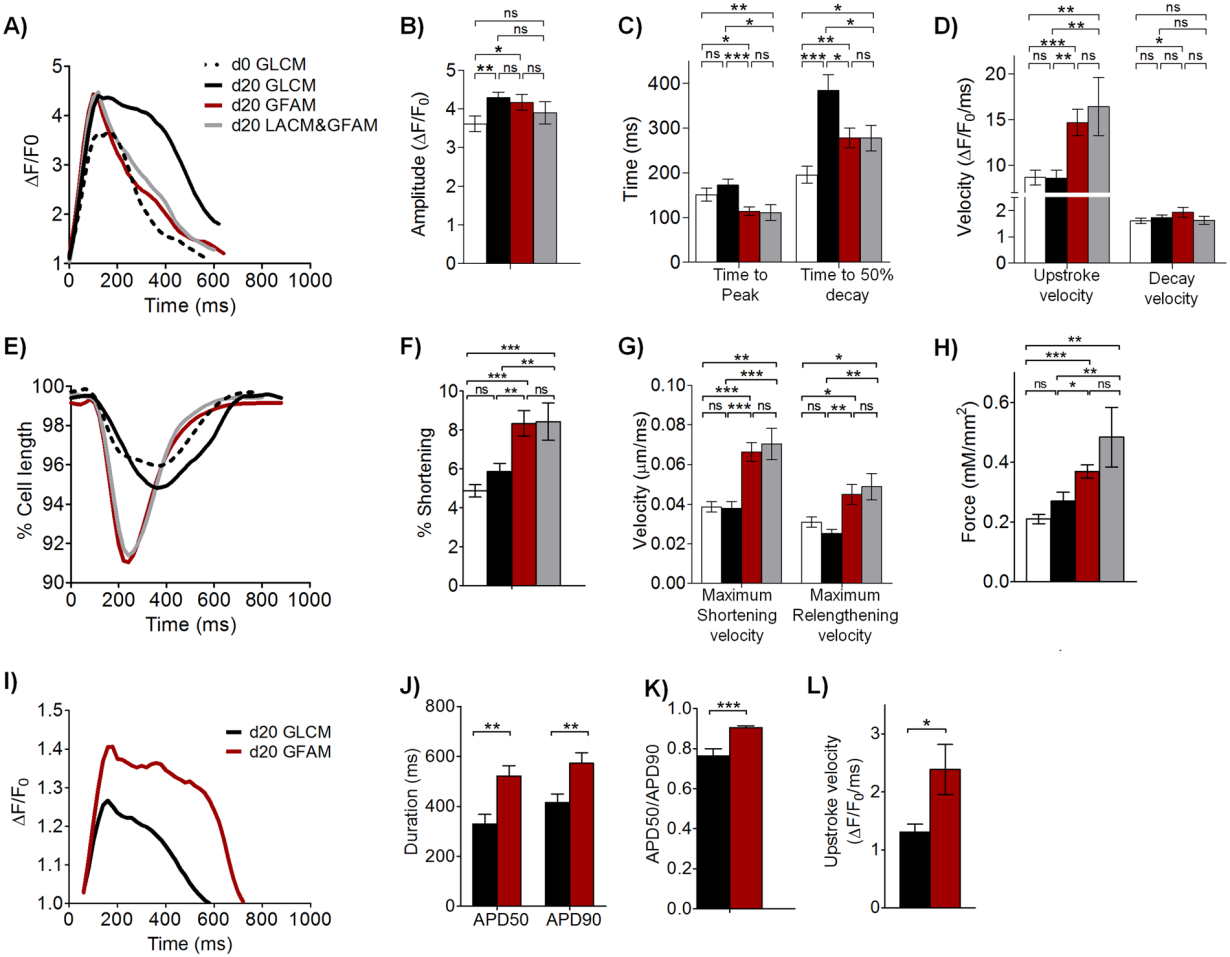
Impact of metabolic manipulation on hiPSC-CM functionality. hiPSC-CMs were analyzed in terms of calcium transients (**A**–**D**), contractile performance (**E**–**H**) and action potential (AP) kinetics (**I**–**L**), before (d0) and after 20 days (d20) of culture in different media. Calcium transient kinetics (**A**–**D**) evaluated with the intracellular calcium indicator Fluo-4 AM: (**A**) Representative calcium transient; (**B)** Calcium transient amplitude (F/F0); (**C**) Time to peak and time to 50% decay; (**D**) Average upstroke and decay velocities. n = 28–35 cells per condition from 3 separate experiments. (**E**) Representative contraction curves, reflecting changes in the percentage of cell length. (**F**) Percentage of shortening; (**G**) Maximum shortening and relengthening velocities. n = 20–35 cells per condition. (**H**) Maximum contractile force generated by hiPSC-CMs in each culture condition. n = 8–17 cells per condition from 3 separate experiments. AP kinetics (I–L) were determined with a voltage-sensitive dye (FluoVolt). (**I**) Changes in the fluorescence intensity of the FluoVolt AP indicator over time. (**J**) AP duration at 50% (APD_50_) and 90% repolarization (APD_90_). (**K**) APD50/APD90. (**L**) AP upstroke velocity. n = 24–26 cells per condition from 3 separate experiments. Data are represented as mean ± SEM. *p < 0.05; **p < 0.01; ***p < 0.001; ns, not significant.

Despite showing similar transient amplitudes (Fig. 6A,B), hiPSC-CMs cultured in GFAM and LACM&GFAM at day 20 presented significantly faster calcium-transient kinetics compared with hiPSC-CMs at days 0 and 20 in GLCM, as indicated by significantly shorter time to peak, time to 50% decay and faster Ca^2+^ transient upstroke and decay velocities (Fig. 6C,D). The faster calcium kinetics observed in GFAM and LACM&GFAM is supported by the high expression of RYR2 and ATP2A2 that are responsible respectively for the release and uptake of Ca^2+^ in the SR (Fig. 4D).

Additionally, compared to day 0 and to cells at day 20 in GLCM, hiPSC-CMs cultured in GFAM and LACM&GFAM exhibited improved contractility with greater fractional shortening (approximately 8.4% of resting length in both LACM&GFAM and GFAM vs. 4.9 (day 0) and 5.9 (GLCM day 20); Fig. 6E,F), faster contractile kinetics (higher shortening and relengthening velocities; Fig. 6G), and greater peak force (0.48 ± 0.22 (LACM&GFAM), 0.37 ± 0.06 mN/mm^2^ (GFAM) vs. 0.21 ± 0.04 (GLCM day 0) and 0.27 ± 0.12 (GLCM day 20); Fig. 6H). Representative movies of calcium and contractility kinetics highlighting the differences between GLCM and GFAM-treated hiPSC-CMs are included in the supplemental material (Movie S1–S2). The significantly lower contractile force generated by the cells in GLCM correlates with the lower expression of contractile and cytoskeletal genes observed in this medium (Fig. 4D). The values of contractile force obtained at day 20 in GFAM (Fig. 6H) were similar to the ones reported after hiPSC-CM maturation with a commercial medium containing T3 hormone (0.43–0.51 mN/mm^2^)^54^.

Importantly, the major functional improvements observed in hiPSC-CMs cultured in GFAM were detected as early as day 10 (Fig. 6 vs. Fig. S7) with no meaningful further improvement in calcium and contractile kinetics observed between 10 and 20 days. This result provides additional evidence that GFAM stimulates a rapid functional maturation of hiPSC-CMs. In contrast, hiPSC-CMs cultured in LACM&GFAM continued to exhibit improvements in fractional shortening, force generation, calcium uptake and shortening velocities from day 10 to 20 (Table S6). These results suggest that GFAM is more efficient than LACM at promoting the rapid functional maturation of hiPSC-CMs. Additionally, no significant differences were observed in calcium and contractile kinetics between cells cultured in GFAM and FAM at day 10 (Fig. S8), suggesting that Gal is not required for the enhancement of hiPSC-CM maturation.

Action potential (AP) durations at 50% and 90% repolarizations (APD50 and APD90), APD50/APD90 and upstroke velocity were significantly higher in GFAM than in GLCM cultures (Fig. 6I–L). These results are consistent with prior studies that report that adult CMs exhibit increased APD90, APD50/90 and maximum upstroke velocity than immature CMs^50, 54, 55^.

Altogether these data demonstrated that GFAM and LACM&GFAM cultures result not only in metabolic, molecular and morphological changes indicative of CM maturation, but also that functionally relevant parameters such as calcium signaling, contraction and action potential are appropriately regulated.

## Discussion

**Figure 7:**
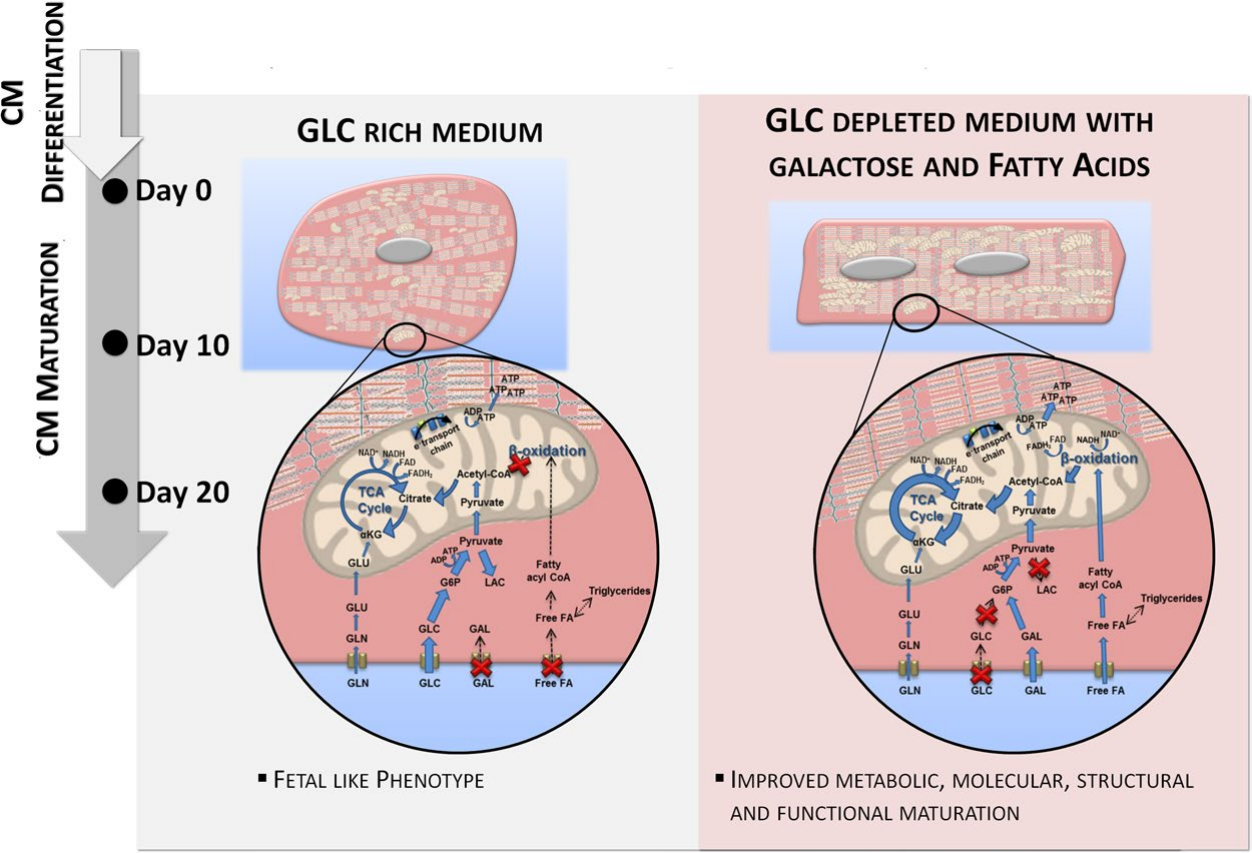
Summary of major findings in this study. The main differences in terms of cell phenotype and metabolism after 20 days of culture in GLCM and GFAM are highlighted.

In this study, we applied a systems-level approach to provide new insights on how the use of different metabolic substrates impacts metabolic, structural and functional maturation of hPSC-CMs. Combining a set of “-omics” tools (metabolomics, fluxomics and transcriptomics) with structural, morphological and functional analyses we show that a glycolytic-to-oxidative metabolic shift can be a cause, rather than a consequence, of the phenotypic alterations characteristic of the CM maturation process. We report for the first time that: i) hiPSC-CMs cultured in medium without Glc supplemented with FA and Gal (GFAM) display improved maturation markers in relation to hiPSC-CMs cultured in standard Glc medium (GLCM; Fig. 7 and Table 1); ii) Gal supplementation ameliorates the oxidative capacity of the cells improving FA oxidation while avoiding lipotoxicity; and iii) hiPSC-CMs cultured in GLCM maintain a fetal-like profile and show features that resemble pathogenic hypertrophy.

**Table 1:** Comparison of structural, functional and metabolic features of hPSC-CMs cultured in GLCM, LACM and GFAM and other approaches reported in the literature with human adult CMs.

Maturation Strategy		Structure			Contractility	Metabolism		Time in culture (day)
		Aspect ratio	Circularity index	Surface area (µm^2^)	Contractile Force	Metabolism	OCR (pmoles/min)	
	GLCM d10	1.8 ± 0.4	0.7 ± 0.1	1359 ± 299	0.3 ± 0.05 (*mN/mm* ^2^)	Glycolytic	—	10
Metabolic substrate manipulation	GLCM d20	2.0 ± 0.5	0.7 ± 0.1	2137 ± 367	0.27 ± 0.12 (*mN/mm* ^2^)	Glycolytic	107 ± 30	20
	FAM d10	5.5 ± 0.9	0.3 ± 0.1	1202 ± 297	0.33 ± 09 (*mN/mm* ^2^)	Oxidative	211 ± 60	10
	LacM d10	3.4 ± 0.7	0.4 ± 0.1	743 ± 245	0.28 ± 0.1 (*mN/mm* ^2^)	Oxidative	—	10
	LACM&GFAM d20	5.2 ± 1.0	0.3 ± 0.1	1093 ± 281	0.48 ± 0.24 (*mN/mm* ^2^)	Oxidative	170 ± 26	20
	GFAM d10	4.2 ± 0.8	0.4 ± 0.1	786 ± 174	0.43 ± 0.23 (*mN/mm* ^2^)	Oxidative	—	10
	GFAM d20	6.5 ± 1.0	0.3 ± 0.1	1021 ± 323	0.4 ± 0.1 (*mN/mm* ^2^)	Oxidative	170 ± 26	20 (35 total)
T3 hormone treatment)^8^		—	0.5 ± 0.0	991 ± 58	12.3 ± 0.7 (*nN/cell*)	—	~169–216	7 (27 total)
Pluricyte Medium (with T3 hormone)^54^		—	—	2169 ± 110	0.5 ± 0.0 (*mN/mm* ^2^)	—	—	20 (33 total)
Electrical stimulation 3D culture^5^		3.7 ± 0.2	—	—	—	—	—	4 (23 total)
Mechanical and electrical stimulation 3D scaffold^7^		—	—	795 ± 46	1.3 ± 0.2 (*mN/mm* ^2^)	—	—	14 (40 total)
Overexpression of let-7^50^		—	—	let-7i: 1110 ± 101 let-7g: 980 ± 95	Let-7i: 11.3 ± 0.9 Let-7g: 9.3 ± 0.7 (*nN/cell*)	—	~150–200	Transduced at day 12 (30 total)
Time in culture^4^		—	0.3 ± 0.0	1716 ± 150	—	—	—	80–120 total
Human Adult CMs^1, 2^		5–9.5:1	–	10212–14418	40–80 (*mN/mm* ^2^) (muscle strips)	Oxidative	—	—

Abbreviations: OCR- Oxygen Consumption Rate; T3 – Triiodothyronine; (—) – Non determined or non reported in the literature; (~) – Approximately.

GFAM culture allows to obtain CMs with a more mature phenotype, characterized by a more energetically efficient oxidative metabolism, transcriptome signatures that more closely resemble adult ventricular CMs, elongated morphologies, organized sarcomeric structures, higher myofibril density and alignment, higher percentage of binucleation, faster calcium-transient kinetics, higher percentage of shortening and contractile force, enhanced AP durations and higher upstroke velocities. Transcriptome analysis revealed that substrate selection induces significant alterations in the expression of key transcriptional regulators and pathways involved in cell metabolism and cardiac development. We showed that hiPSC-CMs have substrate plasticity, being able to immediately adapt to consume other substrates (FA, Gal, Lac, Gln), in the absence of Glc. Rana and colleagues has also previously demonstrated that hiPSC-CMs are capable of utilizing anaerobic or aerobic respiration depending upon the available carbon substrate and showed that hiPSC-CMs rely on OXPHOS for ATP production when cultured in Glc-depleted medium supplemented with Pyr, Gln, Gal and FA^20^. Nevertheless, to date, no studies have quantified the intracellular fluxome and simulated metabolic flux distribution of hiPSC-CMs cultured in distinct carbon sources neither have extensively elucidated how the utilization of these major metabolic substrates modulate structural and functional maturation of hiPSC-CMs as we addressed here. Nonstationary ^13^C-MFA analysis allowed us to confirm quantitatively that hiPSC-CMs present increased metabolic fluxes through FA β-oxidation and TCA cycle and improved ATP production in GFAM, reflecting a highly active OXPHOS.

Even though FA are the main substrates used by adult CMs^16^, Glc-depleted medium supplemented with FA (FAM) induced cell toxicity. Nonetheless, combining FA supplementation with Gal could overcome lipotoxicity by improving oxidative capacity of hiPSC-CMs. Different types of cells (cancer cells, primary fibroblasts and muscle cells) growing in medium in which Glc was replaced by Gal showed enhanced aerobic metabolism^56–58^, due to the induction of an energy deprivation state. Gal is metabolized at a much slower specific rate than Glc due to: i) lower affinity of the transporters (GLUT1 and GLUT4) for Gal compared to Glc and, ii) lower activity of galactokinase, the rate-limiting step in Gal metabolism, comparing to hexokinase, the rate-limiting step in Glc oxidation^59, 60^; forcing cells to start to use OXPHOS to rapidly get sufficient ATP. Even though we observed increased expression of GLUT1 in hiPSC-CMs cultured in GFAM, fluxomic analysis confirmed that the uptake rate of Gal was significantly lower than Glc (30.42 vs. 254.17 nmol/(10^6^cell.h)) when using similar concentrations of both substrates. We confirmed that the flux from Pyr to Lac is negligible and that the majority of Gal is oxidized in TCA cycle and OXPHOS.

It has been described that accumulation of FA leads to augmented intracellular FA derivatives (e.g. fatty acyl CoA, diacylglycerol, and ceramide) that can directly modify cellular structures and activate downstream pathways leading to cellular damage and toxicity^44^. Other studies have shown that saturated FA (palmitate) cause greater lipotoxicity than unsaturated FA (oleate)^40, 61^. Kase *et al*. showed that Gal increases FA oxidation and the formation of neutral lipids that avoid the generation of “lipotoxic” FA derivatives^62^. Additionally, we observed that in GFAM, PPP pathway is highly active while it is negligible in FAM. Since, PPP is one of the main antioxidant cellular defense systems in the cells^63^, we can speculate that Gal also contributes to prevent the detrimental effects of free radical toxicity promoted by OXPHOS. Whether the lipotoxicity observed in FAM derives exclusively from the medium composition itself or from an inadequate adaptation of the cells to oxidative metabolism, still needs to be investigated. In the future it would be interesting to see if a pre-treatment with GFAM before adding FAM would prevent lipotoxicity.

Although this study constitutes a valuable proof of concept that the presence of Gal is important to improve oxidative capacity of the cells, and consequently FA oxidation, several issues remain to be addressed. In particular, investigating different compositions and/or concentrations of key substrates to ultimately deliver an optimal medium formulation for CM maturation would be of major interest for future studies. A Quality by design (QbD) approach^64^, could also be applied to help to rationally define the design space for the identification of the optimal culture medium composition.

We showed that hiPSC-CMs maintained in GLCM during 20 days display a fetal-like CM phenotype and present features of cardiac pathological hypertrophy, namely higher surface area, higher expression of fetal genes, dominant glycolytic energetics, reduced calcium dynamics and contractile force. In accordance, other studies have reported that exacerbated CM pathologies and arrhythmia frequently appear in immature iPSC-CMs within 30 days of culture in non-physiological conditions^19^.

In order to mimic the temporal substrate preference during CM development *in vivo*, we also evaluated the impact of culturing hiPSC-CMs in LACM for 10 days before cultivation in GFAM. Metabolome and transcriptome profiles revealed that LACM improved cells’ oxidative capacity. However, this strategy resulted in higher cell death and reduced structural and functional maturation comparatively to cells cultured for the same period in GFAM. Nonetheless, we showed that combining the additional 10 days of culture in GFAM was sufficient to improve structural and functional maturation of hiPSC-CMs, leading to a phenotype indistinguishable from hiPSC-CMs maintained in GFAM for 20 days. Therefore, we report two valuable strategies to induce metabolism switching and to improve hiPSC-CM maturation, with similar outcomes. The choice of the strategy to use may be dependent on the initial purity state of hiPSC-CM cultures^21^.

Importantly, we confirmed that the benefic effect on metabolic and structural maturation observed in hiPSC-CMs treated with GFAM is reproduced in hESC-CMs, which suggests that this method could be applied to CMs derived from multiple hPSC lines.

During development *in vivo*, CM maturation is regulated by several cues including cell-cell interactions, ECM, soluble factors, mechanical signals and substrate stiffness^1, 2^. To further improve hPSC-CM maturation *in vitro*, this metabolic-based strategy can be combined with other approaches described in literature, such as electrical/mechanical stimulation^5^, co-culture with supporting cells^65^, and medium supplementation (e.g. T3 hormone^8^). Also, it can be adopted for 3D cell culturing approaches^66^, which in combination with the use of environmentally controlled bioreactors^67^ constitute a promising strategy towards scalable production of mature hPSC-CMs.

Overall, by bringing a holistic and quantitative metabolic analysis into the stem cell field, this work not only provides novel insight into the interaction of metabolism and maturation but also lead to the identification and characterization of the most suitable metabolic-based strategy to improve hPSC-CM maturation. This metabolic-based strategy holds technical and economic advantages over the existing protocols due to its scalability, simplicity (a simple medium-exchange procedure) and ease of application (does not require specific equipment or addition of expensive factors/chemicals). Table 1 compares GFAM strategy with other approaches reported in the literature for hPSC-CM maturation that used the same readouts to assess maturation status, namely structure (aspect ratio; circularity index; surface area), functionality (contractile force) and metabolism (metabolic substrate; oxygen consumption rate). The total time of culture is also indicated since it can also affect the culture outcome. It is possible to note that not all approaches use the same readouts to evaluate hPSC-CM maturation, making it difficult to compare the efficiency of the different protocols. Nonetheless, we can conclude that GFAM culture is a competitive and advantageous strategy, capable to induce metabolic, phenotypic and functional maturation of hPSC-CMs in a timely, technically and economically efficient manner, when compared to what has been described in the literature.

Nonetheless, it should be highlighted that although, after GFAM treatment hPSC-CMs present several features more similar to human adult CMs than hPSC-CMs obtained with strategies previously reported (Table 1), hPSC-CMs are still far from becoming structurally and functionally identical to adult CMs. There are key features of the adult CM phenotype which have not yet been ubiquitously observed in culture, including the presence of: i) M-bands, a hallmark of sarcomeric structural maturation^2^, and ii) T-tubules, key structures of the excitation-contraction coupling mechanism in mature CMs that are important for normal Ca^2+^ handling^3^. Additionally, transcriptome analyses revealed that most adrenergic receptors, in particular, the β1(ADRB1)- and β2(ADRB2)-adrenoceptors, recently associated with hiPSC-CM maturation^68^, were not significantly up-regulated in any condition tested. Thus, in future studies, it would be interesting to carefully investigate the inotropic response of GFAM treated cells to β-adrenergic stimulation mediated via canonical β1- and β2-adrenoceptor signaling pathways to evaluate the applicability of these cells in drug discovery.

Even though it has been speculated that a phenotype fully resembling adult CMs might never be attainable in *in vitro* cell culture systems, it is our strong conviction that this study provides a useful contribution for the scientific community to continue pursuing/developing strategies towards the generation of hPSC-CMs with adult-like CM state.

## Electronic supplementary material

Movie S1

Movie S2

Supplementary Info

Supplementary Table 2
