# Supplementary material for: Distinct carbon sources affect structural and functional maturation of cardiomyocytes derived from human pluripotent stem cells
Source: Sci Rep. 2017 Aug 17;7:8590. doi: 10.1038/s41598-017-08713-4 (PMC5561128; doi:10.1038/s41598-017-08713-4)
Supplement: Supplementary file 3 — Supplementary Info [file 41598_2017_8713_MOESM3_ESM.pdf]

Title

Distinct carbon sources affect structural and functional maturation of cardiomyocytes derived from human pluripotent stem cells

Cláudia Correia[1,2], Alexey Koshkin[1,2], Patrícia Duarte[1,2], Dongjian Hu[3,4], Ana Teixeira[1,2,#], Ibrahim Domian[3,4], Margarida Serra[1,2*], Paula M. Alves[1,2*]

[1]iBET, Instituto de Biologia Experimental e Tecnológica, Oeiras, Portugal; [2]Instituto de Tecnologia Química e Biológica António Xavier, Universidade Nova de Lisboa, Oeiras, Portugal; [3]Cardiovascular Research Center, Massachusetts General Hospital, Boston, MA 02114, USA; [4]Harvard Medical School, Boston, MA 02115, USA; Harvard Stem Cell Institute, Cambridge, MA 02138, USA.

[#]Present Address: ETH Zurich, Department of Biosystems Science and Engineering, Mattenstrasse 26, 4058 - Basel, Switzerland

*Corresponding authors: Paula Marques Alves and Margarida Serra, Animal Cell Technology Unit, iBET, Instituto de Biologia Experimental e Tecnológica and Instituto de Tecnologia Química e Biológica, Apartado 12, 2781-901 Oeiras, Portugal (Phone: +351 21 446 94 31; FAX:+351 21 442 11 61; e-mail: marques@ibet.pt; mserra@itqb.unl.pt); website: http://tca.itqb.unl.pt.

Supporting Information

Supplemental Figures

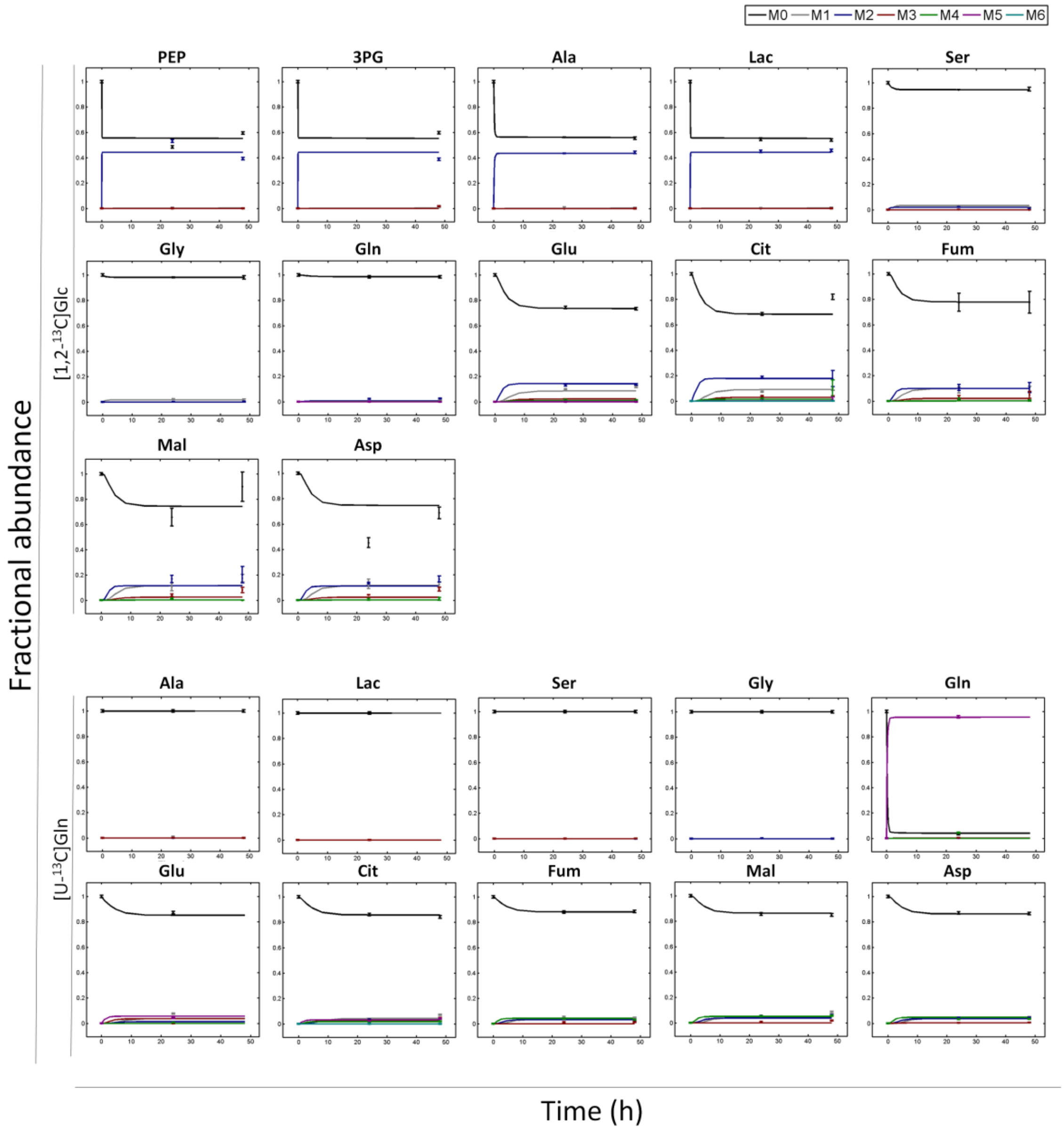

Fig. S1. Experimental and simulated intracellular ^{13}C-labelling dynamics in GLCM condition using [1,2-^{13}C]GLC and [U-^{13}C]GLN. Circle markers correspond to GC-MS measurements corrected for natural isotope abundance. Lines correspond to fitted MIDs from nonstationary 13C-MFA flux estimation of parallel labelling experiments.

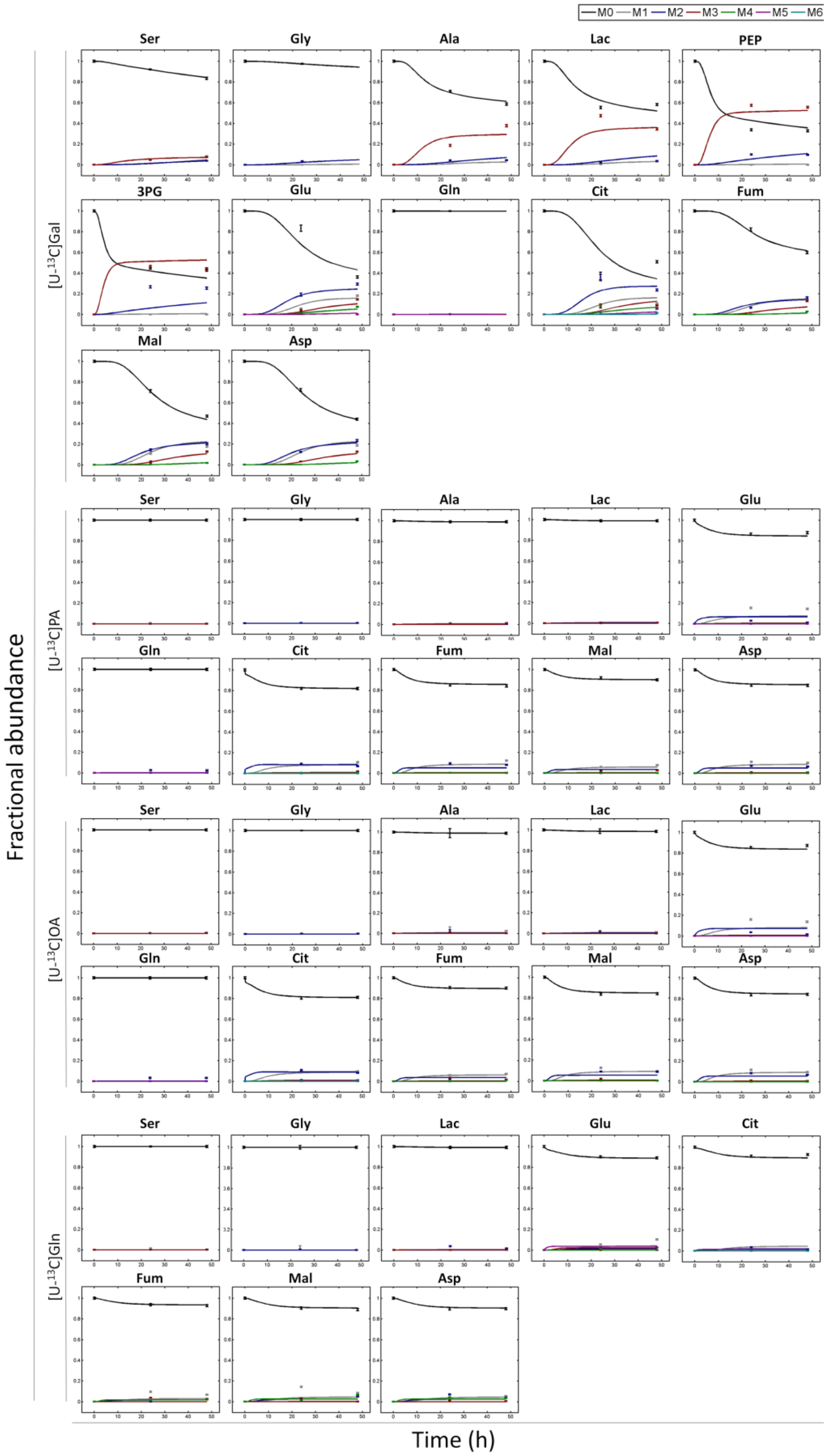

Fig. S2. Experimental and simulated intracellular ^{13}C-labelling dynamics in GFAM condition using [U-^{13}C]GAL, [U-^{13}C]PA, [U-^{13}C]OA and [U-^{13}C]Gln. Circle markers correspond to GC-MS

measurements corrected for natural isotope abundance. Lines correspond to fitted MIDs from nonstationary 13C-MFA flux estimation of parallel labelling experiments.

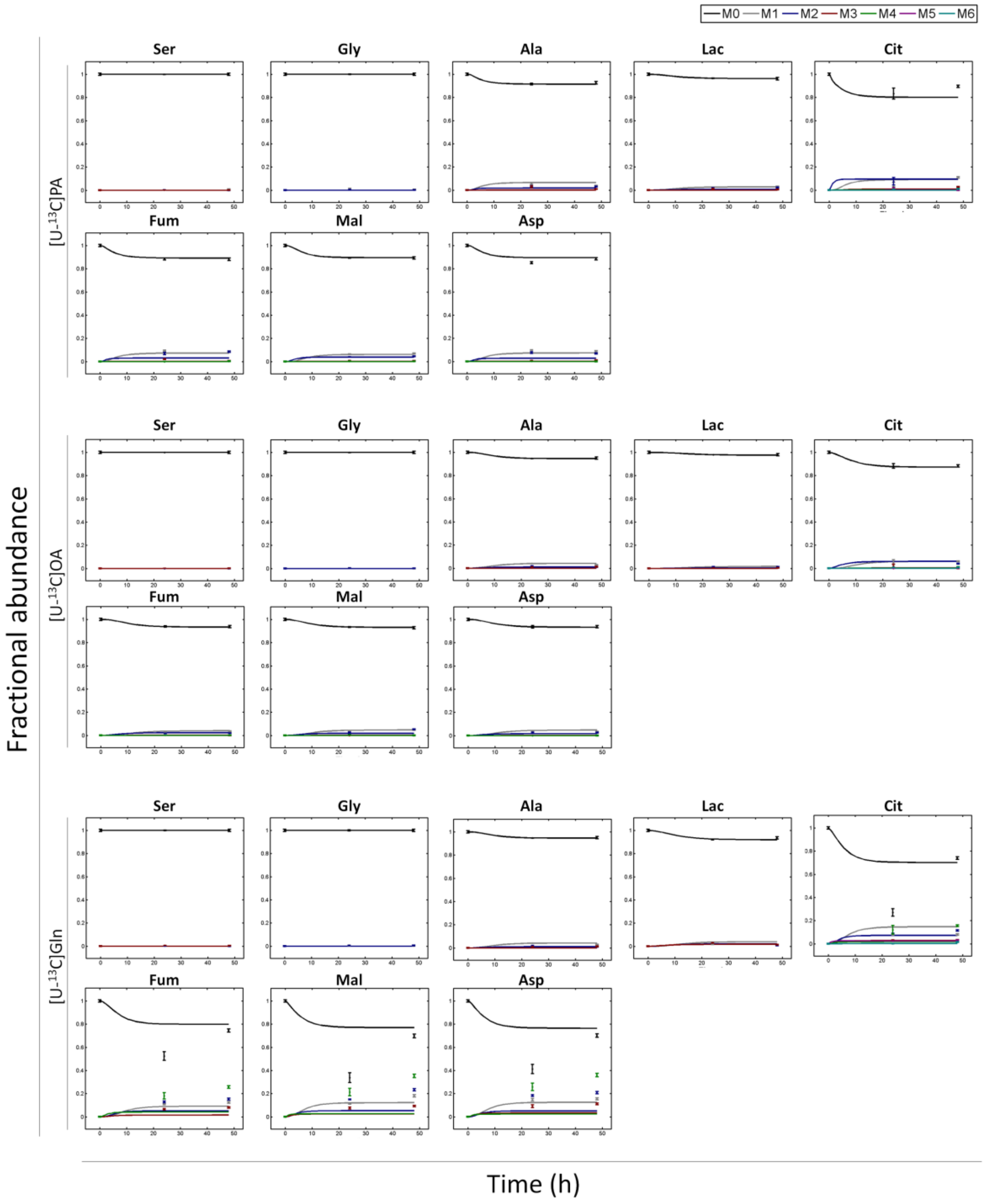

Fig. S3. Experimental and simulated intracellular ^{13}C-labelling dynamics in FAM condition using [U-^{13}C]PA, [U-^{13}C]OA and [U-^{13}C]Gln. Circle markers correspond to GC-MS measurements corrected for natural isotope abundance. Lines correspond to fitted MIDs from nonstationary 13C-MFA flux estimation of parallel labelling experiments.

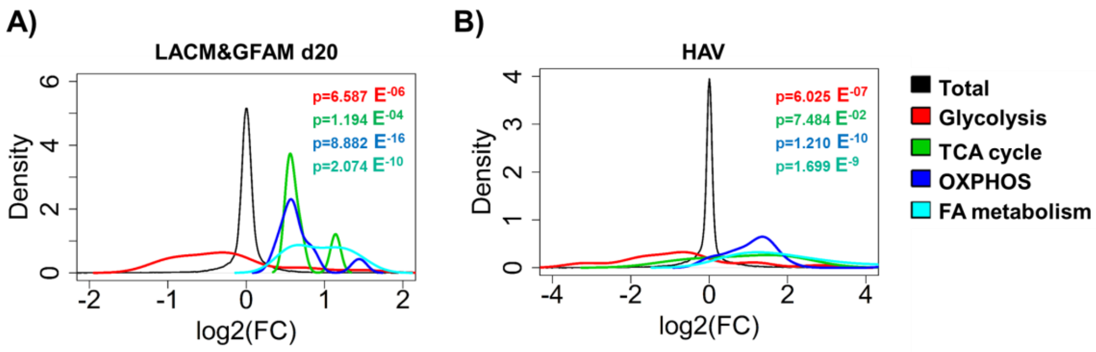

Fig. S4. Density Plots using R generated with fold change expression of genes from representative metabolic categories for LACM&GFAM d20 (A) and HAV (B). X axis indicates $\log_2$ fold change in gene expression. Black line indicates expression of all genes. Coloured lines toward the left and right side of the black line indicate down-regulation and up-regulation of pathways, respectively.

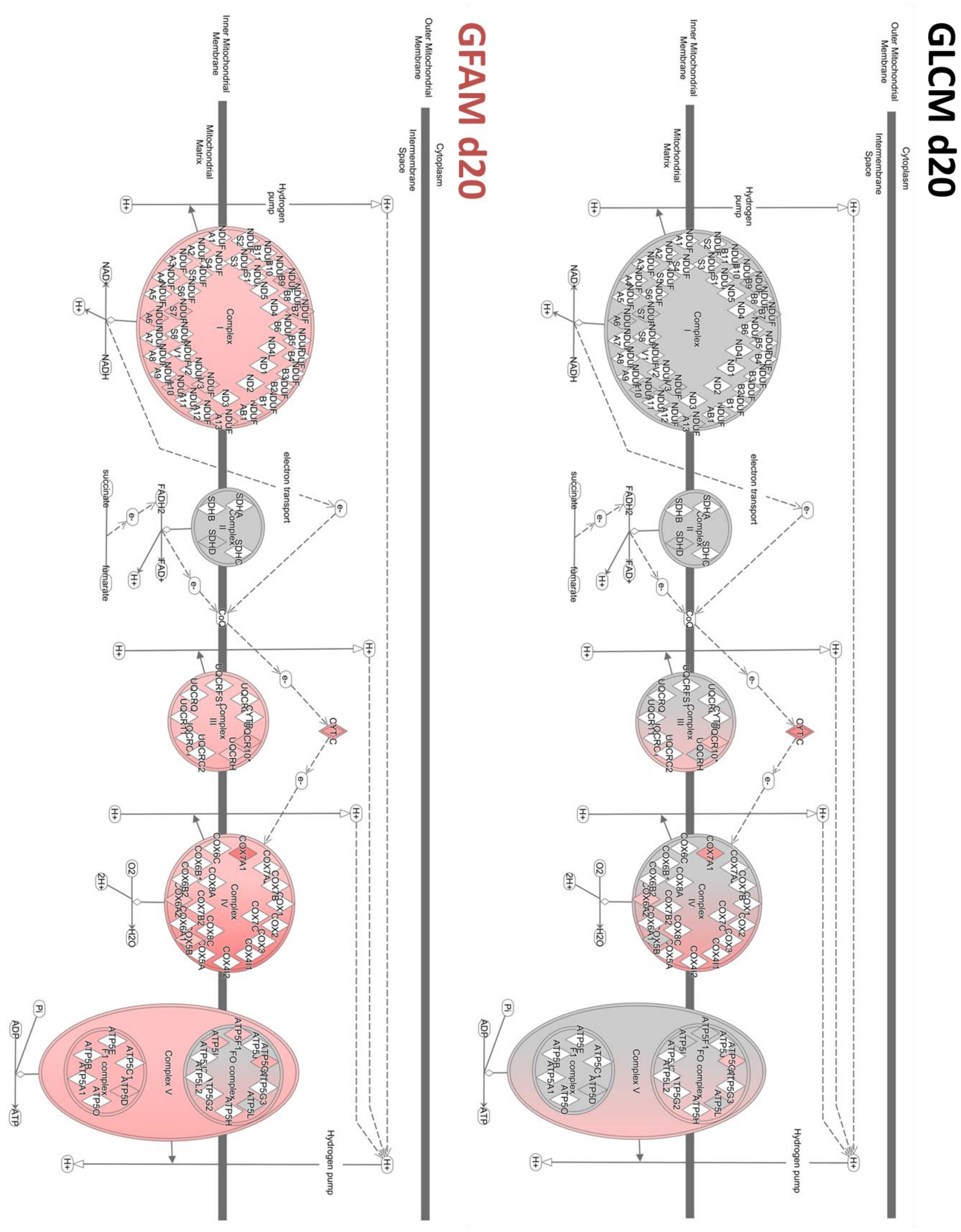

Fig. S5. Schematic representation of the expression levels of OXPHOS related genes provided by IPA for GLCM and GFAM cultures at day 20. Up-regulated genes are highlighted in red.

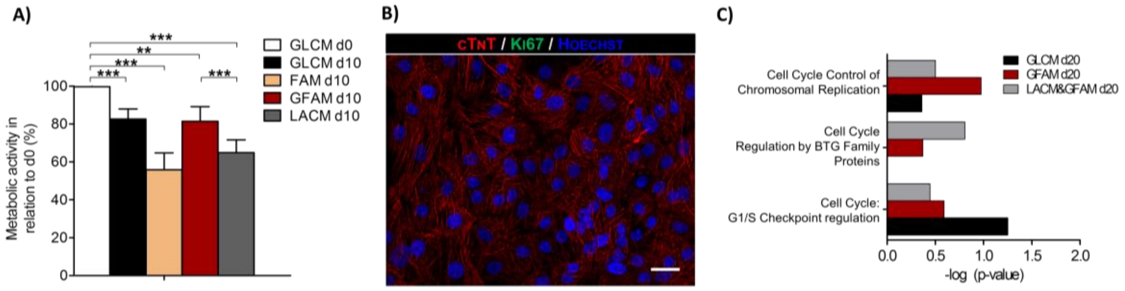

Fig. S6. Impact of culture media composition on metabolic activity and proliferative capacity. (A) Metabolic activity at day 0 and day 10 in all conditions was assessed with PrestoBlue. (B) Representative image of hPSC-CM immunostained for cardiac troponin-T (cTnT) revealing the absence of Ki67^{+} cells (green). Nuclei (blue) were stained with Hoechst 33342. Scale bars=30 µm. (C) Adjusted P-value determined by IPA reflecting non-statistically significant enrichment of cell-cycle related pathways in all culture condition.

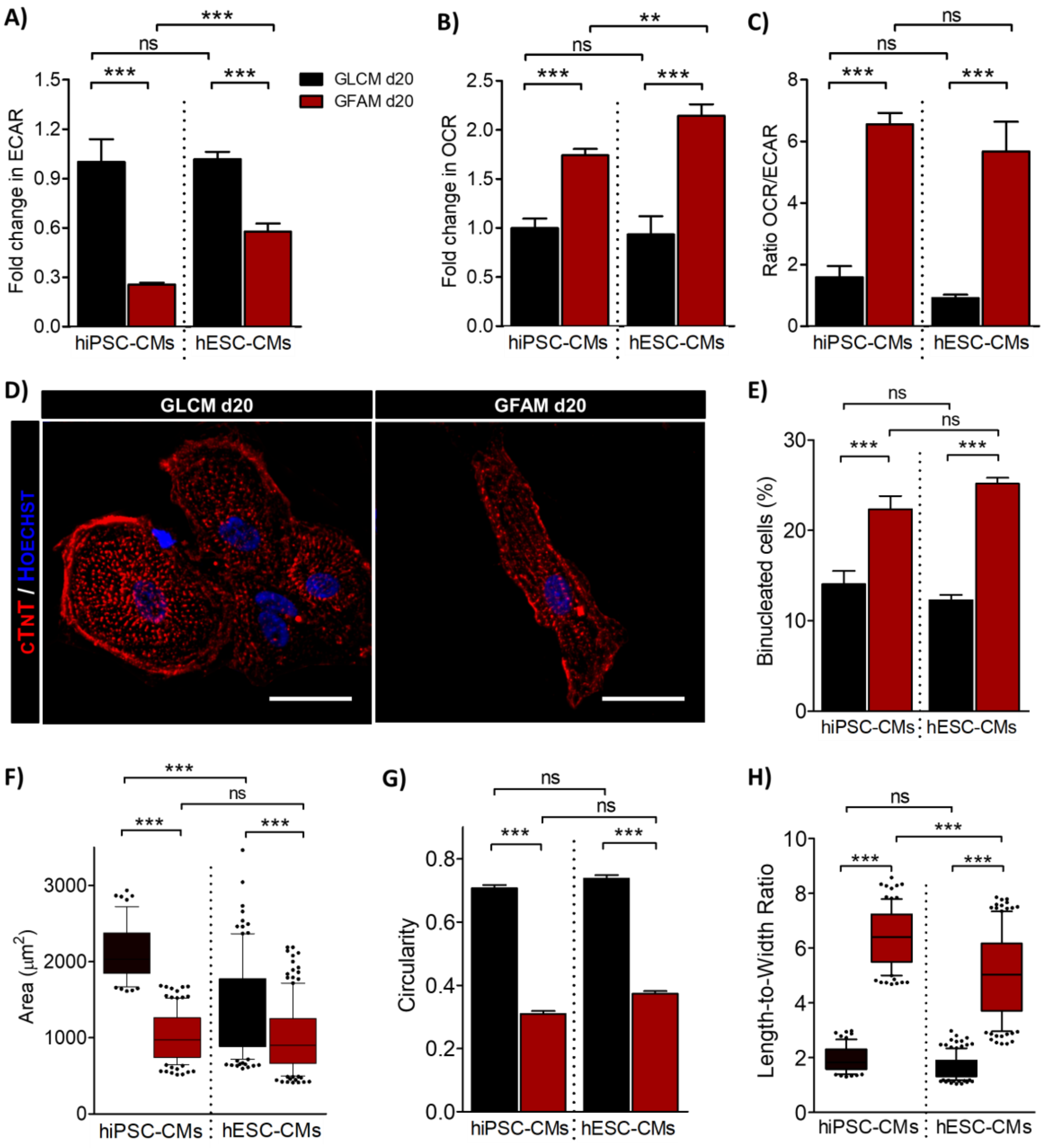

Fig. S7. Impact of GFAM treatment on hESC-CM metabolism and structure. hESC-CMs maintained in GLCM and GFAM for 20 days were compared in terms of cellular metabolism and structure between each other and with hiPSC-CMs cultured also in GLCM and GFAM. (A) Extracellular acidification rate (ECAR). (B) Basal oxygen consumption rate (OCR). (C) OCR/ECAR ratio reflecting the relative contribution of OXPHOS over glycolysis for energy generation. (n>20 per condition of 3 different cell batches). (D) Representative images of hPSC-CM immunostained for cardiac troponin-T (cTnT, red). Nuclei (blue) were stained with Hoechst 33342. Scale bars=30 μm. (E) Percentage of binucleated hiPSC-CMs in GLCM and GFAM (n>20 per condition). (F-H) Cell structure characterization in terms of cell area (F), circularity index (G), length-to-width ratio (H). n>70 cells per condition of 3 separate experiments. Data are represented as mean±SD.

*p<0.05; **p<0.01; ***p<0.001; ns, not significant.

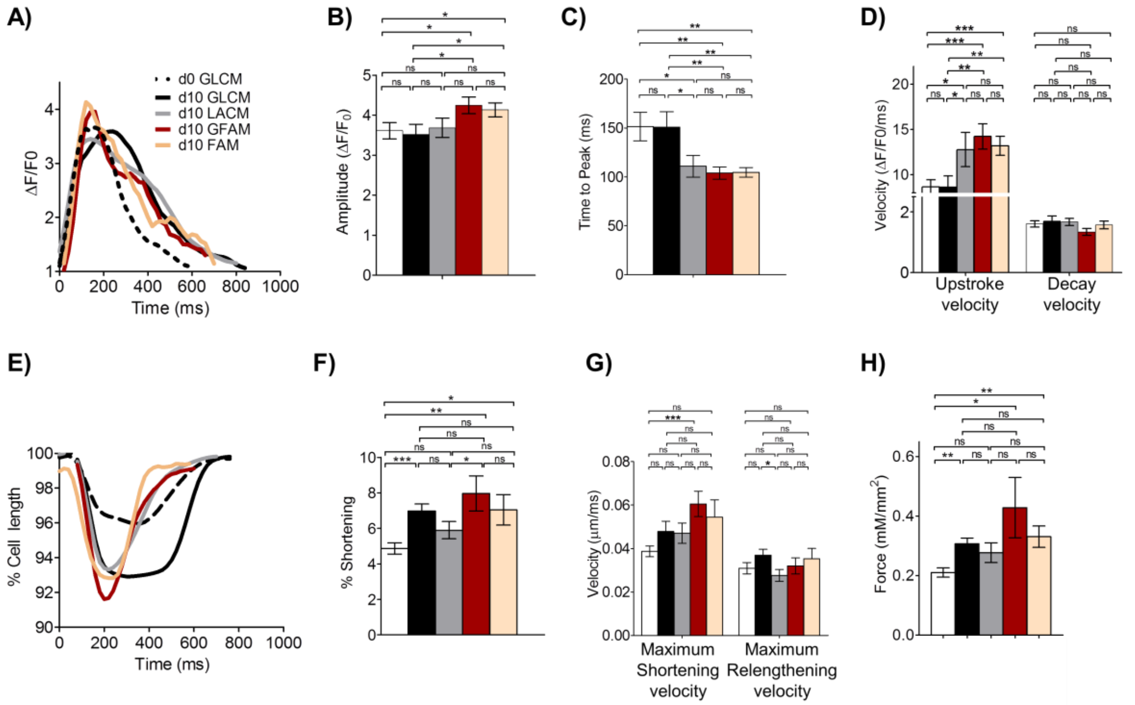

Fig. S8. Impact of metabolic manipulation in hiPSC-CM functionality. hiPSC-CMs were analyzed in terms of calcium signaling profiles (A-D) and contractile performance (E-H), before (white) and after 10 days of culture in different culture medium (GLCM-black, LACM -grey, GFAM-red and FAM). Calcium transient kinetic was evaluated by loading the hiPSC-CMs with the intracellular calcium indicator Fluo-4 AM: (A) Representative calcium transients. F/F0: change of fluorescence signal over background florescence; (B) Calcium transient amplitude (F/F0); (C) Time to peak. (D) Average upstroke and decay velocities. n = 24-35 cells per condition of 3 separate experiments. Contractile kinetics for each culture condition is described in: (E) Representative contraction curves for each culture condition, reflecting changes in the percentage of cell length. (F) Percentage of shortening; (G) Average shortening and relengthening velocities. n = 15-22 cells per condition of 3 separate experiments. (H) Maximum contractile force generated. n = 8-17 cells per condition of 3 separate experiments. Data are represented as mean±SEM. *p<0.05; **p<0.01; ***p<0.001; ns, not significant.

Table S1. Metabolic network reactions and simulated fluxes determined by nonstationary ^{13}C-MFA. Flux values are shown in units of nmol/(10^6cell.h). Negative flux values indicate that net direction is reverse to the direction indicated. The lower bound (LB) and upper bound (UB) of the 95% confidence intervals determined for each flux are also displayed.

		GLCM d20						FAM d10						GFAM d20					
	Equation	Value		LB		UB		Value		LB		UB		Value		LB		UB	
**v1**	G6P <-> F6P	220.5	[	150	;	347.1	]	0	[	0	;	0	]	3.9	[	1.8	;	5.4	]
**v2**	F6P -> FBP	242.9	[	165	;	326.2	]	0	[	0	;	0	]	21.6	[	15.9	;	22.3	]
**v3**	FBP <-> DHAP + GAP	242.9	[	165	;	326.2	]	0	[	0	;	0	]	21.6	[	15.9	;	22.3	]
**v4**	DHAP <-> GAP	242.9	[	165	;	326.2	]	0	[	0	;	0	]	21.6	[	15.9	;	22.3	]
**v5**	GAP <-> 3PG	497.1	[	338.4	;	660.4	]	0	[	0	;	0	]	52	[	38.1	;	54	]
**v6**	3PG <-> PEP	496.9	[	334.3	;	658.4	]	0	[	0	;	0	]	49.7	[	36.8	;	53.2	]
**v7**	PEP -> Pyr.c	496.9	[	334.3	;	658.4	]	0	[	0	;	0	]	49.7	[	36.8	;	53.2	]
**v8**	Pyr.c <-> Pyr.m	5.6	[	1.4	;	8.6	]	109.2	[	106.3	;	109.3	]	72.4	[	56.3	;	75.6	]
**v9**	G6P -> P5P + CO2	33.7	[	0	;	50.2	]	0	[	0	;	0	]	26.5	[	18.8	;	29.2	]
**v10**	P5P + P5P <-> GAP + S7P	11.2	[	0	;	16.7	]	0	[	0	;	0	]	8.8	[	6.3	;	9.7	]
**v11**	S7P + GAP <-> E4P + F6P	11.2	[	0	;	16.7	]	0	[	0	;	0	]	8.8	[	6.3	;	9.7	]
**v12**	E4P + P5P <-> GAP + F6P	11.2	[	0	;	16.7	]	0	[	0	;	0	]	8.8	[	6.3	;	9.7	]
**v13**	Pyr.c <-> Lac	491.4	[	336.4	;	651.4	]	0	[	0	;	0	]	0	[	0	;	0	]
**v14**	Pyr.c <-> Ala	0	[	-7.8	;	14	]	0	[	0	;	0	]	-11	[	-12.3	;	-9.1	]
**v15**	Pyr.m -> AcCoA.m + CO2	5.6	[	2.6	;	8.6	]	30.3	[	30.2	;	30.3	]	72.4	[	54.8	;	75.3	]
**v16**	OAA.m + AcCoA.m -> Cit.m	14.1	[	6.3	;	21.6	]	55.4	[	55.3	;	55.4	]	88.5	[	66.5	;	92.2	]
**v17**	Cit.m <-> AKG + CO2	14.1	[	6.4	;	20.3	]	22.1	[	22.1	;	22.2	]	88.5	[	66.7	;	92.1	]
**v18**	AKG -> SucCoA + CO2	14.7	[	6.9	;	27.2	]	43.3	[	43.2	;	43.4	]	95.7	[	71.1	;	100.1	]
**v19**	SucCoA <-> Suc	14.7	[	6.9	;	27.2	]	43.3	[	43.2	;	43.4	]	95.7	[	71.1	;	100.1	]
**v20**	Suc <-> Fum.m	14.7	[	6.8	;	26.7	]	45.7	[	45.6	;	45.9	]	95.7	[	72	;	99.4	]
**v21**	Fum.m <-> Mal.m	14.7	[	4	;	22.8	]	52.1	[	52.0	;	52.2	]	95.7	[	72.2	;	99.3	]
**v22**	Mal.m <-> OAA.m	14.1	[	6.3	;	21.4	]	-23.5	[	-23.7	;	-23.4	]	88.5	[	66.4	;	91.5	]
**v23**	Mal.c -> Pyr.c + CO2	0	[	0	;	4.2	]	108.9	[	108.7	;	109.5	]	7.2	[	4.7	;	8.3	]
**v24**	Pyr.m + CO2 -> OAA.m	0	[	0	;	0	]	78.9	[	78.4	;	79.4	]	0	[	0	;	0.5	]
**v25**	Gln <-> Glu	1	[	0.4	;	1.7	]	3.8	[	3.6	;	3.9	]	2.6	[	1.9	;	2.7	]

**v26**	AKG <-> Glu	-0.6	[	-6.4	;	-0.3	]	-21.2	[	-22.8	;	-20.2	]	-7.2	[	-8.4	;	-4.8	]
**v27**	Asn <-> Asp	0	[	0	;	0	]	0	[	0	;	0	]	0	[	0	;	0	]
**v28**	3PG -> Ser	0.2	[	0	;	13.2	]	0	[	0	;	0	]	2.3	[	0.7	;	3.8	]
**v29**	Ser -> Pyr.c	0	[	0	;	8.3	]	0.3	[	0.2	;	0.3	]	4.5	[	1.1	;	7	]
**v30**	Ser <-> Gly + C1	-1.8	[	-4.1	;	0	]	-2.4	[	-2.4	;	-2.4	]	-0.3	[	-2	;	0	]
**v31**	Glu <-> Pro	0	[	-4.4	;	3.5	]	-10.5	[	-10.5	;	-10.5	]	-1.4	[	-4.1	;	1.4	]
**v32**	Val + CO2 -> Suc + CO2 + CO2	0	[	0	;	0.1	]	0	[	0	;	0	]	0	[	0	;	0.5	]
**v33**	Ile + CO2 -> Suc + AcCoA.m + CO2	0	[	0	;	0.1	]	0	[	0	;	0	]	0	[	0	;	0.5	]
**v34**	Leu + CO2 -> AcCoA.m + AcCoA.m + AcCoA.m + CO2	2.2	[	1.2	;	3.3	]	0	[	0	;	0	]	1.6	[	0.1	;	2.6	]
**v35**	Thr -> AcCoA.m + Gly	0	[	0	;	1.6	]	3.9	[	3.9	;	4.0	]	0.3	[	0.1	;	0.6	]
**v36**	Phe -> Tyr	0	[	0	;	0.1	]	0	[	0	;	0	]	0	[	0	;	0.4	]
**v37**	Tyr -> Fum.m + AcCoA.m + AcCoA.m + CO2	0	[	0	;	0.1	]	6.4	[	6.3	;	6.4	]	0	[	0	;	0.4	]
**v38**	Met + Ser + CO2 -> Suc + Cys.snk + CO2 + C1	0	[	0	;	0	]	2.4	[	2.4	;	2.5	]	0	[	0	;	0.4	]
**v39**	Lys -> CO2 + CO2 + AcCoA.m + AcCoA.m	0.9	[	0	;	1.8	]	0	[	0	;	0	]	0	[	0	;	1.5	]
**v40**	His -> Glu + C1	1.8	[	0	;	4.2	]	0	[	0	;	0	]	0.3	[	0	;	2	]
**v41**	Arg -> Glu + Urea.snk	59.8	[	0	;	Inf	]	0	[	0	;	0	]	3.6	[	0	;	4.1	]
**v42**	Glu + CO2 -> Arg	64	[	0.7	;	Inf	]	0	[	0	;	0	]	2.7	[	0	;	2.9	]
**v43**	Glc.ext -> G6P	254.2	[	178	;	326.1	]	0	[	0	;	0	]	0	[	0	;	0.2	]
**v44**	Lac.ext <-> Lac	-491.4	[	-651.4	;	-336.4	]	0	[	0	;	0	]	0	[	0	;	0	]
**v45**	Ala.ext <-> Ala	0	[	-14	;	7.8	]	0	[	0	;	0	]	11	[	9.1	;	12.3	]
**v46**	Gln.ext -> Gln	1	[	0.4	;	1.7	]	3.8	[	3.8	;	3.8	]	2.6	[	1.9	;	2.7	]
**v47**	Glu.ext <-> Glu	2	[	-1.2	;	5.8	]	6.9	[	6.9	;	6.9	]	2.1	[	-0.2	;	4.4	]
**v48**	Ser.ext <-> Ser	-1.9	[	-9.7	;	0.8	]	0.3	[	0.2	;	0.4	]	1.9	[	0.7	;	3.0	]
**v49**	Gly.ext <-> Gly	1.8	[	-1.6	;	4.1	]	-1.5	[	-1.6	;	-1.4	]	-0.1	[	-0.6	;	1.6	]
**v50**	Pro.ext <-> Pro	0	[	-3.5	;	4.4	]	10.5	[	10.5	;	10.5	]	1.4	[	-1.4	;	4.1	]
**v51**	Val.ext -> Val	0	[	0	;	0.1	]	0	[	0	;	0	]	0	[	0	;	0.5	]
**v52**	Ile.ext -> Ile	0	[	0	;	0.1	]	0	[	0	;	0	]	0	[	0	;	0.5	]
**v53**	Leu.ext -> Leu	2.2	[	1.2	;	3.3	]	0	[	0	;	0	]	1.6	[	0.1	;	2.6	]
**v54**	Thr.ext -> Thr	0	[	0	;	1.6	]	3.9	[	3.9	;	4	]	0.3	[	0.1	;	0.6	]
**v55**	Phe.ext -> Phe	0	[	0	;	0.1	]	0	[	0	;	0.	]	0	[	0	;	0.4	]

**v56**	Tyr.ext -> Tyr	0	[	0	;	0.1	]	6.4	[	6.3	;	6.4	]	0	[	0	;	0.3	]
**v57**	Met.ext -> Met	0	[	0	;	0	]	2.4	[	2.4	;	2.5	]	0	[	0	;	0.4	]
**v58**	Lys.ext -> Lys	0.9	[	0	;	1.8	]	0	[	0	;	0	]	0	[	0	;	1.5	]
**v59**	His.ext -> His	1.8	[	0	;	4.2	]	0	[	0	;	0	]	0.3	[	0	;	2	]
**v60**	Arg.ext <-> Arg	-4.2	[	-7	;	-1.3	]	0	[	0	;	0	]	0.9	[	-2.4	;	3.8	]
**v61**	Asp <-> OAA.c	-0.6	[	-1.8	;	-0.3	]	0	[	0	;	0	]	0	[	-1	;	0	]
**v62**	Cit.c -> OAA.c + AcCoA.c	0	[	0	;	0.1	]	33.3	[	33.2	;	33.3	]	0	[	0	;	1.8	]
**v63**	CO2.ext <-> CO2	-5.9	[	-90	;	Inf	]	-132.1	[	-132.2	;	-132.1	]	-287.8	[	-303.4	;	-213.4	]
**v64**	AcCoA.c -> FA.ext	0	[	0	;	0.1	]	33.3	[	33.2	;	33.3	]	0	[	0	;	1.8	]
**v65**	AP.ext -> AP.c	0	[	0	;	0	]	1.9	[	1.9	;	1.9	]	0.7	[	0.5	;	0.7	]
**v66**	AP.m -> 8 x AcCoA.m	0	[	0	;	0	]	8x0.6	[	8x0.6	;	8x0.7	]	8x0.7	[	8x0.5	;	8x0.7	]
**v67**	AO.ext -> AO.c	0	[	0	;	0	]	1.7	[	1.7	;	2.3	]	0.6	[	0.5	;	0.7	]
**v68**	AO.m -> 9 x AcCoA.m	0	[	0	;	0	]	9x0.4	[	9x0.4	;	9x1.5	]	9x0.6	[	9x0.5	;	9x0.6	]
**v69**	Gal.ext -> G6P	0	[	0	;	0	]	0	[	0	;	0	]	30.4	[	22	;	32.1	]
**v70**	Cit.c -> Cit.snk	0	[	0	;	1.9	]	0	[	0	;	0	]	0	[	0	;	1.5	]
**v71**	Mal.m <-> Mal.c	0.6	[	0.2	;	4.4	]	75.6	[	75.3	;	75.7	]	7.2	[	4.5	;	8.3	]
**v72**	0*Mal.c -> Mal.s	3.9	[	0	;	100	]	0	[	0	;	6.4	]	0.5	[	0	;	100	]
**v73**	0*Mal.m -> Mal.s	96.1	[	0	;	100	]	100	[	93.6	;	100	]	99.5	[	0	;	100	]
**v74**	Mal.s -> Sink	100	[	100	;	100	]	100	[	100	;	100	]	100	[	100	;	100	]
**v75**	0*Fum.c -> Fum.s	14	[	6.2	;	21.6	]	0	[	0	;	0.2	]	32.2	[	28.9	;	36.3	]
**v76**	0*Fum.m -> Fum.s	86	[	78.4	;	93.8	]	100	[	99.8	;	100	]	67.8	[	63.7	;	71.1	]
**v77**	Fum.s -> Sink	100	[	100	;	100	]	100	[	100	;	100	]	100	[	100	;	100	]
**v78**	Mal.c <-> Fum.c	0	[	0	;	0	]	0	[	0	;	0	]	0	[	0	;	0	]
**v79**	OAA.c <-> Mal.c	-0.6	[	-1.9	;	-0.2	]	33.3	[	33.3	;	33.3	]	0	[	-1	;	0	]
**v80**	Asp.ext <-> Asp	-0.6	[	-1.8	;	-0.3	]	0	[	0	;	0	]	0	[	-1	;	0	]
**v81**	0*Cit.c -> Cit.s	0	[	0	;	100	]	0	[	0	;	100	]	83.5	[	0	;	100	]
**v82**	0*Cit.m -> Cit.s	100	[	0	;	100	]	100	[	0	;	100	]	16.5	[	0	;	100	]
**v83**	Cit.s -> Sink	100	[	100	;	100	]	100	[	100	;	100	]	100	[	100	;	100	]
**v84**	Cit.c <-> Cit.m	0	[	-2	;	0	]	-33.3	[	-33.3	;	-33.2	]	0	[	-1.6	;	0	]
**v85**	AP.c -> AP.m	0	[	0	;	0	]	0.6	[	0.6	;	0.7	]	0.7	[	0.5	;	0.7	]

**v86**	AP.c -> AP.snk	0	[	0	;	0	]	0.4	[	0.4	;	1.5	]	0.6	[	0.5	;	0.6	]
**v87**	AO.c -> AO.m	0	[	0	;	0	]	1.3	[	1.2	;	1.3	]	0	[	0	;	0.1	]
**v88**	AO.c -> AO.snk	0	[	0	;	0	]	1.3	[	1.3	;	3.5	]	0	[	0	;	0.1	]

Abbreviations: G6P. glucose-6-phosphate; F6P. fructose-6-phosphate; FBP. fructose-1.6-bisphosphate; DHAP. Dihydroxyacetone phosphate; GAP. glyceraldehyde 3-phosphate; 3PG. 3-Phosphoglyceric acid; PEP. phosphoenolpyruvate; Pyr. pyruvate; P5P. pentose-5-phosphate; CO2. carbon dioxide; S7P. sedoheptulose-7-phosphate; E4P. erythrose 4-phosphate; Lac. lactate; Ala. alanine; AcCoA. acetyl-CoA; OAA. oxaloacetate; Cit. citrate;αKG. α-ketoglutarate; Suc. succinate; Fum. fumarate; Mal. malate; Glu. glutamate; Pro. proline; Val. valine; Ile. isoleucine; Leu. leucine; Thr. threonine; Phe. phenylalanine; Tyr. tyrosine. Met. methionine; Ser. serine; Cys. cyteine; lys. lysine; His. histidine; Arg; arginine; Gln. glutamine; Asp. aspartate; FA. fatty acid; PA. palmitic acid; AO. oleic acid; c. cytosolic; m. mitochondrial; s. sink.
Fumarate and succinate were considered as symmetric molecules during flux estimation. Compartmentalization of Pyr, Fum, Mal, Cit, AcCoA, OAA, AO and AP pools were taken in account in the model. Sink pools were considered for metabolites that could not be balanced.
Balanced metabolite pools: G6P, F6P, FBP, DHAP, GAP, 3PG, PEP, Pyr.c, Pyr.m, P5P, CO 2, S7P, E4P, Lac, Ala, AO.c, AO.m, AP.c, AP.m, AcCoA.c, AcCoA.m, OAA.c,OAA.m, Pyr.c, Pyr.m, Cit.c, Cit.m, AKG, SucCoA, Suc, Fum.c, Fum.m, Mal.c,Mal.m, Gln, Glu, Asn, Asp, Ser, Gly, C1, Pro, Val, Ile, Leu, Thr, Phe, Tyr, Met, Lys, His, Arg.
Unbalanced metabolite pools: AO.ext, AP.ext, Ao.snk, Ap.snk, Cit.snk, FA.ext, CO 2.ext, Glc.ext, Gal.ext Lac.ext, Ala.ext, Gln.ext, Glu.ext, Asp.ext, Asn.ext, Ser.ext, Gly.ext, Pro.ext, Val.ext, Ile.ext, Leu.ext, Thr. ext, Phe.ext, Tyr.ext, Met.ext, Lys.ext, His.ext, Arg.ext, Cys.snk, Urea.snk.

Table S3 - Pearson correlation coefficients between conditions represented in Figure 3.F, respective 95% and 99% confidence intervals (95%CI and 99%CI) and statistical significance.

	Correlation coefficient	95%CI		99%CI		p-value (two-tailed)
		Lower	Upper	Lower	Upper	
**HAV vs LACM&GFAM**	0.524	0.503	0.545	0.496	0.551	< 0.001
**HAV vs. GFAM d20**	0.526	0.505	0.546	0.498	0.553	< 0.001
**HAV vs. GLCM d0**	0.221	0.193	0.248	0.185	0.257	0.112
**HAV vs. GLCM d20**	0.238	0.211	0.265	0.202	0.273	0.086
**LACM&GFAM vs. GFAM d20**	0.987	0.986	0.988	0.986	0.988	< 0.001
**LACM&GFAM vs. GLCM d0**	0.861	0.853	0.868	0.851	0.87	< 0.001
**LACM&GFAM vs. GLCM d20**	0.868	0.861	0.875	0.858	0.877	< 0.001
**GFAM d20 vs. GLCM d0**	0.884	0.878	0.89	0.875	0.892	< 0.001
**GFAM d20 vs. GLCM d20**	0.88	0.873	0.886	0.871	0.888	< 0.001
**GLCM d0 vs. GLCM d20**	0.984	0.983	0.985	0.983	0.985	< 0.001

Table S4 - Euclidean distances between closest neighboring samples represented in PCA plots (Figure 3G and 4A), calculated considering the space defined by the first (PC1) and second (PC2) components.

	Euclidean distance (Figure 3G)	Euclidean distance (Figure 4A)
HAV vs LACM&GFAM d20	11.55±2.50	81.83±7.92
HAV vs. GFAM d20	11.08±0.94	82.95±3.83
HAV vs. GLCM d0	14.85±2.18	128.04±3.65
HAV vs. GLCM d20	14.34±1.08	82.37±7.35
LACM&GFAM d20 vs. GFAM d20	1.61±0.09	4.38±3.25
LACM&GFAM d20 vs. GLCM d0	8.72±1.04	96.42±0.51
LACM&GFAM d20 vs. GLCM d20	8.19±0.76	29.71±5.49
GFAM d20 vs. GLCM d0	7.94±0.16	93.13±2.66
GFAM d20 vs. GLCM d20	7.24±0.02	26.81±7.66
GLCM d0 vs. GLCM d20	1.32±0.06	67.49±5.63

Table S5 - Pearson correlation coefficients between conditions represented in Figure 4.B, respective 95% and 99% confidence intervals (95%CI and 99%CI) and statistical significance.

	Correlation coefficient	95%CI		99%CI		p-value (two-tailed)
		Lower	Upper	Lower	Upper	
**HAV vs LACM&GFAM d20**	0.885	0.879	0.891	0.877	0.893	< 0.001
**HAV vs. GFAM d20**	0.878	0.871	0.884	0.869	0.886	< 0.001
**HAV vs. GLCM d0**	0.763	0.761	0.784	0.757	0.788	< 0.001
**HAV vs. GLCM d20**	0.840	0.831	0.848	0.829	0.851	< 0.001
**LACM&GFAM vs. GFAM d20**	0.994	0.994	0.994	0.994	0.994	< 0.001
**LACM&GFAM vs. GLCM d0**	0.943	0.94	0.946	0.939	0.947	< 0.001
**LACM&GFAM vs. GLCM d20**	0.957	0.955	0.959	0.954	0.96	< 0.001
**GFAM d20 vs. GLCM d0**	0.953	0.95	0.956	0.949	0.956	< 0.001
**GFAM d20 vs. GLCM d20**	0.960	0.958	0.962	0.957	0.963	< 0.001
**GLCM d0 vs. GLCM d20**	0.965	0.963	0.967	0.962	0.968	< 0.001

Table S6. Comparison of contractility kinetics and contractile force generated by cells cultivated in LACM&GFAM at d10 and d20. Data are represented as mean±SD. *p<0.05; **p<0.01.

	LACM&GFAM d10	LACM&GFAM d20	Statistical Analysis
**Force (mM/mm^2)**	0.28±0.10	0.48±0.22	*
**% Shortening**	5.90±2.17	8.42±3.30	*
**Maximum shortening velocity (µm/ms)**	0.05±0.02	0.07±0.03	*
**Maximum relengthening velocity (µm/ms)**	0.03±0.01	0.05±0.02	**
